# At the world's edge: Reconstructing diet and geographic origins in medieval Iceland using isotope and trace element analyses

**DOI:** 10.1002/ajpa.23973

**Published:** 2019-12-13

**Authors:** Joe W. Walser, Steinunn Kristjánsdóttir, Darren R. Gröcke, Rebecca L. Gowland, Tina Jakob, Geoff M. Nowell, Chris J. Ottley, Janet Montgomery

**Affiliations:** ^1^ Curator of Artifact Collections National Museum of Iceland Hafnarfjörður Iceland; ^2^ Department of History and Philosophy University of Iceland Reykjavík Iceland; ^3^ Department of Earth Sciences Durham University Durham UK; ^4^ Department of Archaeology Durham University Durham UK

**Keywords:** bioarchaeology, diet, isotopes, palaeopathology, provenance

## Abstract

**Objectives:**

A multi‐isotope study was conducted on individuals buried at Skriðuklaustur monastery (AD 1493–1554) to investigate their geographic origins and dietary composition. Comparative material from individuals excavated from Skeljastaðir, an inland farm site was also analyzed.

**Materials and methods:**

Bone collagen was extracted from 50 humans (Skriðuklaustur and Skeljastaðir) and 25 animals (Skriðuklaustur) and analyzed for δ^13^C, δ^15^N, and δ^34^S. Dental enamel samples from 31 individuals (Skriðuklaustur) were also analyzed for ^87^Sr/^86^Sr, δ^18^O, δ^13^C, and trace elements (Pb, Sr, Zn, Ba).

**Results:**

The mean value determined from individuals from Skriðuklaustur (*n* = 36) was δ^13^C = −18.7 ± 0.8‰, δ^15^N = 12.8 ± 1.1‰, and δ^34^S = 9.0 ± 1.6‰, whereas at Skeljastaðir (*n* = 14), it was δ^13^C = −20.5 ± 0.8‰, δ^15^N = 7.8 ± 0.9‰, and δ^34^S = 9.4 ± 1.6‰. At Skriðuklaustur, human dental enamel samples (*n* = 31) provided a ^87^Sr/^86^Sr range of 0.7060–0.7088, δ^18^O_phosphate_ from 13.9 to 16.1‰ and δ^13^C_carbonate_ from −16.6 to −12.9‰. Inferred drinking water (δ^18^O_dw_) values range from −12.3 to −8.9‰. Sr concentrations range from 25.8 to 156.7 ppm, Ba from 0.11 to 0.81 ppm, Zn from 43.8 to 145.8 ppm, and Pb from 0.13 to 9.40 ppm.

**Discussion:**

A combination of results indicates that the people from Skriðuklaustur were born in Iceland, but some lived inland during childhood while others lived closer to the coast. Since Skriðuklaustur was a hospital, these individuals may have sought medical treatment at the monastery. The δ^13^C and δ^15^N values determined from bone collagen indicate that the people residing at Skriðuklaustur consumed a diet high in marine protein, while those residing at Skeljastaðir exhibit values more consistent with terrestrial resources.

## 1 INTRODUCTION

**Figure 1:**
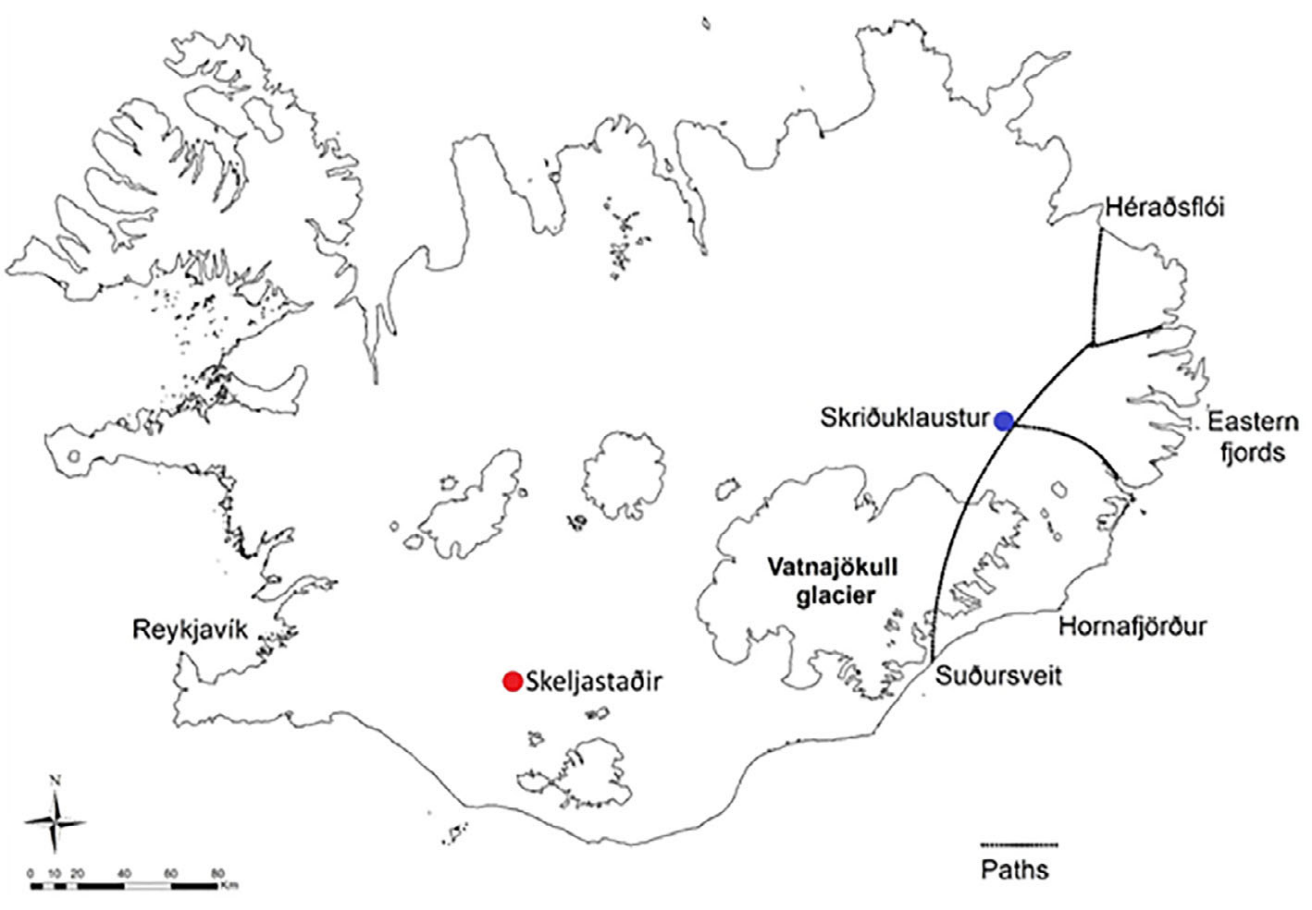
A map of Iceland depicting the locations of Skriðuklaustur and Skeljastaðir. The major routes of trade and travel to and from Skriðuklaustur are also shown (^©^Kristjánsdóttir and Gunnarsdóttir)

Recent excavations at the site of Skriðuklaustur (AD 1493–1554), located in an inland valley in eastern Iceland, demonstrated that this monastery functioned not only as a place of scholarly work and monastic activities, but also a place where the community could seek medical care and treatment (Figure 1). Although oral history suggests the presence of post‐Reformation burials marked by gravestones at Skriðuklaustur, these graves have never been located. Radiocarbon dating and other archaeological dating methods demonstrated that the site was in use during the monastic period, specifically between AD 1493 and 1554 (Kristjánsdóttir, 2012, pp. 153–154). Historical and archaeological evidence has revealed specialized medical knowledge, surgical tools, diverse medicinal plants and herbs, and imported objects and food, indicative of extensive involvement with foreign trade and monastic networks. In 2002–2012, the skeletal remains of around 300 individuals were excavated from the site, and these presented with a vast array of pathological conditions, including infectious diseases, traumatic injuries, and congenital anomalies (Kristjánsdóttir, 2008; Kristjánsdóttir, 2011; Kristjánsdóttir, 2012; Kristjánsdóttir & Collins, 2011). Comparative material was sought from a subset of skeletons excavated from a cemetery belonging to a single, inland farm site at Skeljastaðir in the Þjórsárdalur valley in southern Iceland (Figure 1). This site is around 60 km from the coast; however, there are numerous lakes and rivers with freshwater fish in the region. The farm was occupied earlier than Skriðuklaustur, originally believed to be from around ~AD 1000 until at least AD 1104 when the enormous eruption of Hekla occurred, likely leading to the abandonment of the site (Gestsdóttir, 2014). Radiocarbon dating of a subset (*n* = 7) of the human skeletal remains suggested a date range between AD 890 and 1220 (Sveinbjörnsdóttir et al., 2010). However, some human activity and animal grazing persisted in the valley at least until another eruption occurred in AD 1300 (Dugmore et al., 2007). In 1939, 63 skeletons were excavated from the early Christian cemetery at Skeljastaðir, although only 56 skeletons are available for study today (Gestsdóttir, 2014; Steffensen, 1943). This site was selected due to the extensive research conducted on it (e.g., archaeology, palaeoclimatology, multiple stable isotope analyses, palaeopathology), its well‐preserved material, sample size, and geographic location. Both are inland sites without direct access to coastal resources; Skeljastaðir is located in the highlands, a mostly uninhabitable volcanic desert, while Skriðuklaustur is located in a mountainous area.

The geographic origin of the individuals buried at Skriðuklaustur is unknown; these individuals may have traveled to the monastery during pilgrimages, for trade or to seek treatment from elsewhere in the country or from abroad. The aim of this research was to undertake a multi‐isotope study of human and animal remains from the site to establish the origins of the individuals buried there: did the monastery serve predominantly the local community, or had the inhabitants traveled from further afield? Considering the monastic context, this research also sought to evaluate dietary composition and the possible dietary differences between men, women, and children, or between religious and secular individuals buried at the monastery. Stable isotope analyses of δ^13^C, δ^15^N, and δ^34^S were obtained from samples of human and animal bone collagen and of δ^13^C_,_ δ^18^O, and ^87^Sr/^86^Sr from human dental enamel samples. Trace element (Zn, Ba, Sr, and Pb) concentrations were also measured in the dental enamel samples to establish baselines for Icelandic humans and to attempt to further examine geographic provenance. Regarding Skeljastaðir, the previous isotopic study of δ^13^C and δ^15^N in bone collagen samples (*n* = 13) demonstrated a diet predominately derived from terrestrial resources (see Sveinbjörnsdóttir et al., 2010). Since δ^34^S values have been used in mobility and palaeodietary studies to differentiate between freshwater, marine, and terrestrial resources, we aimed to refine these interpretations by evaluating possible freshwater fish consumption at Skeljastaðir. The other previous analysis conducted on the Skeljastaðir assemblage identified only one non‐Icelandic migrant according to strontium isotope analysis (see Gestsdóttir & Price, 2003). The current study investigates the utility of δ^34^S analyses to improve interpretations of local versus nonlocal origins within Iceland given the potential relative homogeneity of strontium isotopes deriving from the predominantly igneous basalt bedrock. Finally, we wished to compare the diet between individuals buried in these two very different settlement types (monastic and farm). Isotopic analyses have not been conducted on humans or fauna from Skriðuklaustur prior to this research and this is the first in‐depth multi‐isotope study of its kind undertaken on a skeletal collection dated to the Late Medieval Period from Iceland. This research thereby provides unique data that considers the link between diet and geographic provenance from an isotopic perspective. It provides previously unavailable Icelandic human and animal isotope baselines and comparative data from the Late Medieval Period that will be useful for future research concerning Iceland and other locales in the North Atlantic.

## 2 CURRENT EVIDENCE FOR DIET AND MOBILITY IN HISTORICAL ICELAND

Iceland is located just below the Arctic Circle. Despite the challenging, subpolar climate, people were proficient in supplying themselves with a variety of food from the beginning of the Settlement period (AD 871 ± 2) (Karlsson, 2000). During the Middle Ages, sheep, dairy products, and fish, especially dried fish, were the primary staples of the diet and a substantial proportion of terrestrial foods were acidified, soured, fermented, or salt preserved (Mehler, 2011). It was common to consume bone marrow as well, particularly from sheep long bones (Outram, 2003). Other protein sources included cow, pig, duck, goose, and various sea birds as well as eggs (Mehler, 2011; Svanberg & Ægisson, 2012). Domestic fowl (*Gallus gallus sp*.) have been only very rarely identified from Icelandic archaeological sites prior to the 17th century (Hamilton‐Dyer, 2010). Additionally, an important trade economy was in place that provided the country with access to a variety of nonlocal food products. For example, one historical record dated AD 1567 states that for over 50 years traders from Bremen, Germany imported tons of flour, salt, beer, vinegar, grains, and bread, although most of these commodities did not become dietary staples until the 20th century (DI 15, nr. 12; Gísladóttir, 1999; Mehler, 2011). Despite ample local production, fish and meats were also imported to Iceland and exchanged for goods, such as walrus ivory and refined sulfur (Mehler, 2011).

Although barley cultivation was practiced, the growing season was short and challenging, thereby limiting the production yield (Mehler, 2011; Mooney & Guðmundsdóttir, in press; Svanberg & Ægisson, 2012). Frequent volcanic eruptions, epidemics, and the climatic changes that occurred with the Little Ice Age (~13th–19th century AD) also presented significant challenges to successful cultivation in Iceland (Dugmore & Vesteinsson, 2012; McGovern, Harrison, & Smiarowski, 2014). While some scholars suggest that plant‐based foods were a minor dietary component (McGovern et al., 2014; Mehler, 2011), subsistence gardening may have been more important to the historical diet than previously believed (Kristjánsdóttir, Larsson, & Åsen, 2014). In the monastic gardens, in addition to the import and cultivation of barley, a wide variety of plants, such as nettles (*Urtica dioica*), angelica (*Angelica archangelica*), field garlic (*Allium oleraceum*), valerian (*Valeriana officinalis*), yarrow (*Achillea millefolium*), and greater plantain (*Plantago major*), were grown for dietary, utility, and medicinal purposes (Kristjánsdóttir et al., 2014; Larsson & Lundquist, 2010). According to the Sagas, ancient Icelanders even brewed mead and beer using imported malt and local barley (Gísladóttir, 1994, p. 124; Mehler, 2011) and wine was produced from foraged crowberries (Jóhannesson, 2014, p. 307). Around 30 taxa of foraged wild plants also played a small, but important role as dietary resources, particularly in times of hardship (Svanberg & Ægisson, 2012) (see Table 1).

**Table 1:** Examples of some of the most commonly consumed edible wild plants in historical Iceland (see Gísladóttir, 1999; Mehler, 2011; Svanberg & Ægisson, 2012)

Common name	Icelandic name	Scientific name	Plant part	Purpose
Common silverweed	Tágamura	*Potentilla anserine*	Root	Subsistence
Common horsetail	Klóelfting	*Equisetum arvense*	Root	Subsistence
Garden angelica	Ætihvönn	*Angelica archangelica*	Root, leaves	Subsistence, medicinal
Scurvy grass	Skarfakál	*Cochlearia officinalis*	Root, leaves	Subsistence, medicinal
Common sorrel	Túnsúra	*Rumex acetosa*	Leaves	Subsistence
Iceland moss	Fjallagrös	*Cetraria islandica*	Moss	Subsistence, medicinal
Bilberry	Aðalbláberjalyng	*Vaccinium myrtillus*	Berries	Subsistence, seasoning
Bog bilberry	Bláberjalyng	*Vaccinium uliginosum*	Berries	Subsistence, seasoning
Crowberry	Krækilyng	*Empetrum nigrum*	Berries	Subsistence, seasoning
Dulse	Söl	*Palmaria palmata*	Seaweed	Subsistence
Carrageen moss	Fjörugrös	*Chondrus crispus*	Seaweed	Subsistence
Wild thyme	Blóðberg	*Thymus praecox*	Leaves, flowers	Tea, seasoning
Caraway	Kúmen	*Carum carvi*	Seeds	Seasoning
Common juniper	Einir	*Juniperus communis*	Berries	Seasoning
Common butterwort	Lyfjagras	*Pinguicula vulgaris*	Leaves	Seasoning, medicinal
Mountain avens	Holtasóley	*Dryas octopetala*	Leaves, flowers	Tea

People residing in high latitude regions generally attain much of their protein from animal sources (Cordain et al., 2000). For example, isotopic studies conducted on the Greenland Norse revealed that in the early period of settlement approximately 20% of the diet was marine‐based, increasing to 50–80% in later periods (Arneborg et al., 1999; Nelson, Heinemeier, Lynnerup, Sveinbjörnsdóttir, & Arneborg, 2012). A study of proto‐Inuit individuals revealed δ^15^N values reached as high as 20‰ due to the frequent consumption of whales and seals (Coltrain, Hayes, & O'Rourke, 2004). Seals were a notable meat and fat source in historical Iceland, but whales were only consumed if stranded or beached (Mehler, 2011; Outram, 2003; Riddell, 2015).

In Iceland, historical and archaeological evidence suggests that subsidiary traditions primarily came from Scandinavia; however, the longstanding tradition of gathering and consuming seaweed (Dulse) likely came from Celtic settlers during the Settlement Period (Sigurðsson, 1988). Isotopic studies have demonstrated that the consumption of seaweed can raise δ^13^C values, resulting in a dietary signature that appears to be more marine‐based (Schulting, Vaiglova, Crozier, & Reimer, 2017). Marine isotope signals could also be introduced in humans who consume terrestrial animals that graze upon seaweed (Schulting & Richards, 2009). In historical Iceland, sheep that were kept near seaweed beaches regularly grazed upon seaweeds and farmers also deliberately fed dried seaweed to their sheep for up to 18 weeks a year (Hallsson, 1964). Fishmeal, made of Icelandic herring, is often mixed with hay to supplement protein for sheep, particularly ewes (Sveinbjörnsson & Einarsson, 1998). Although it is not clear when the use of fishmeal as sheep fodder began, the steady exploitation of herring started as early as the 9th century AD (McGovern, Perdikaris, Einarsson, & Sidell, 2006). The heavy dietary reliance on meat from fish‐eating sheep in historical Iceland must therefore be considered as a potential confounding factor in isotopic signals determined from human bone collagen.

### 2.1 Dietary reconstruction in Iceland: Carbon (δ^13^C_carbonate_ and δ^13^C_collagen_), nitrogen (δ^15^N_collagen_), and sulfur (δ^34^S_collagen_) isotopes

Pagan and early Christian bone samples selected from all over Iceland by Sveinbjörnsdóttir et al. (2010) produced δ^15^N_collagen_ values between 6.5 and 15.5‰ and δ^13^C_collagen_ values as ranging between −20.3 and −16.4‰, with most of the data in the range of −20 to −18‰. Recently, Price and Gestsdóttir (2018) reported mean δ^13^C_collagen_ values (*n* = 37) as −20‰ and mean δ^15^N_collagen_ values as 10‰, while Sayle et al. (2016) reported δ^34^S values between 5.5 and 14.9‰ in human samples from northern Iceland. Price and Gestsdóttir (2018) measured δ^13^C_carbonate_ isotopes from enamel samples (*n* = 116) from pagan and early Christian graves throughout Iceland and obtained a mean δ^13^C_carbonate_ value of −15.3‰ ± 0.95 (range of −17.3 to −12.4‰). Previous isotopic studies conducted on Icelandic material demonstrated overlaps between δ^13^C values in freshwater and marine biota, as well as between δ^15^N values of terrestrial herbivores and freshwater fish species. As a result, it is often difficult to differentiate between these dietary components in Iceland using δ^13^C and δ^15^N alone (Ascough et al., 2014). δ^34^S values, however, have been successfully used to differentiate between dietary components in Icelandic animal and human remains once local baselines are established. Sayle et al. (2016) noted in their study at Mývatn in northern Iceland that higher δ^13^C and δ^34^S values indicate marine protein as a component of regular diet, while higher δ^13^C and lower δ^34^S values indicate freshwater protein. The study also noted that higher δ^13^C and δ^15^N versus lower δ^34^S values indicates the consumption of a large proportion of freshwater protein, while lower δ^13^C and δ^15^N versus higher δ^34^S values may indicate migration from another place, likely a coastal area where δ^34^S values are altered by the sea spray effect (Sayle et al., 2016). This combination of isotopic analyses enabled the researchers to differentiate between terrestrial, freshwater, and marine dietary signals as well as to identify possible migrants and examine aspects of livestock trade and animal husbandry (see Sayle et al., 2016). However, due to differences in geology and volcanic activity, it must be noted that δ^34^S values measured in skeletal material from northern Iceland may not necessarily entirely correspond with δ^34^S values determined from skeletal material from southern Iceland. Isotopic value ranges established from Icelandic material (i.e., animals, water, and geology) to date are presented in Table 2.

**Table 2:** Isotope value ranges from both modern flora, seawater, freshwater, geology, and archaeological and modern fauna from Iceland

Material	δ^13^C‰	δ^15^N‰	δ^34^S‰	Source
Birds	−23.6 to −6.9	−4.9 to 16.4	−5.3 to 13.6	Ascough et al. (2014) and Wang and Wooller (2006)
Freshwater fish	−16.0 to −7.9	3.1 to 8.5	−4.3 to −0.2	Ascough et al. (2014) and Sayle et al. (2016)^a^
Aquatic plants	−16.9 to −11.5	−16.0 to 4.3	—	Ascough et al. (2014) and Wang and Wooller (2006)
Terrestrial plants	−30.9 to −20.4	−12.4 to 6.5	—	Ascough et al. (2014); Skrzypek, Paul, and Wojtun (2008); and Wang and Wooller (2006)
Terrestrial herbivores	−22.5 to −20.3	−1.5 to 5.9	−1.0 to 13.9	Ascough et al. (2014) and Sayle et al. (2013)
Marine fish/mammals	−16.3 to −13.5	12.1 to 14.5	12.4 to 17.5	Sayle et al. (2013)
Freshwater and geology	—	—	−2.0 to 10.0	Sayle et al. (2013)^b^
Seawater	—	—	Mean 20.3	Nehlich (2015)

a Unpublished data by Ascough, reported in Sayle et al. (2016).

b Sayle et al. (2013), and references therein, report the range of δ^34^S‰ in Icelandic geology (volcanic/basaltic) and water sources (ground, flood, river, and spring water).

### 2.2 Residential mobility in Iceland: Strontium (^87^Sr/^86^Sr) and oxygen (δ^18^O) analysis of dental enamel

Price and Gestsdóttir (2006) analyzed strontium isotope (^87^Sr/^86^Sr) ratios in dental enamel from 83 pre‐Christian individuals buried across the country, identifying at least 32 nonlocal migrants, that is, those having ^87^Sr/^86^Sr values above ~0.7092 ‐ the value for rain and seawater ‐ which defines the upper ^87^Sr/^86^Sr end member for the basaltic biosphere of Iceland. Recently, Price and Gestsdóttir (2018) expanded this study by bringing the total sample size to 127 individuals and by analyzing ^87^Sr/^86^Sr, δ^18^O, and δ^13^C from dental enamel and δ^13^C and δ^15^N from bone collagen. Icelandic inhabitants ^87^Sr/^86^Sr ratios range between 0.7055 and 0.7092 (Price & Gestsdóttir, 2018). Icelandic geologic and bioavailable strontium isotope baselines (^87^Sr/^86^Sr) are presented in Table 3. Price et al. (2015) noted that bioavailable strontium isotope ratios in Iceland are higher than those from whole rock (~0.7030–0.7037; Sigmarsson et al., 1992) due to the sea spray that occurs over much of the country. Such a phenomenon has been observed in other basaltic and particularly high‐rainfall environments such as the Isle of Skye (Evans, Montgomery, & Wildman, 2009) and Hawaii (Capo, Stewart, & Chadwick, 1998). The swamping of geological ^87^Sr/^86^Sr by atmospheric deposition via rainwater and marine seasplash and spray has also been observed in maritime granitic and sandstone island biospheres such as the Western and Northern Isles of Scotland where the geology contributes ^87^Sr/^86^Sr values considerably higher than 0.7092 (Montgomery et al., 2014; Montgomery, Evans, & Cooper, 2007). Price et al. (2015) and Price and Gestsdóttir (2006, 2018) also suggested that variations in ^87^Sr/^86^Sr may reflect dietary differences between inland (values closer to 0.7030 reflecting the basalt geology) and coastal (values closer to 0.7092 reflecting the input of seawater) dwelling individuals. Strontium isotope ratios close to those of seawater in archaeological coastal and island populations are often coupled with unusually high strontium concentrations and such a combination may be explained by the high concentration of diet‐derived strontium from marine plant‐based resources and the extent of aerial sea spray deposition (Montgomery et al., 2007; Montgomery & Evans, 2006). Cultural culinary and husbandry practices, such as using seaweed for food, fodder, or fertilizer, grazing animals on coastal floodplains or preserving food in sea salt, may thus elevate animal and human strontium concentrations and provide a dietary ^87^Sr/^86^Sr value of 0.7092 even when humans are not consuming fish or marine mammal meat which are both low in bioaccessible strontium (unless bones are consumed) compared to plant‐based foods (Montgomery et al., 2007; Montgomery & Evans, 2006).

**Table 3:** Strontium ratios (^87^Sr/^86^Sr) determined in bedrock, grass grown on volcanic soil, modern barley, and seawater and from modern and archaeological faunal dental enamel. The sheep samples were collected from inland sheep that did not graze upon seaweed, which can raise strontium isotope ratios (Price, Frei, & Naumann, 2015)

Enamel sample	^87^Sr/^86^Sr	*n*	Source	Material	^87^Sr/^86^Sr	Source
Archaeological cattle	0.7042	2	Price et al. (2015)	Bedrock	0.7030–0.7037	Price et al. (2015) and Sigmarsson, Condomines, and Fourcade (1992)
Archaeological pig	0.7042	1	Price et al. (2015)	Grass (volcanic soil)	0.7030–0.7040	Åberg (1995)
Modern redshank bird	0.7057	5	Evans and Bullman (2009)	Barley	0.7068	Price et al. (2015)
Modern sheep	0.7059–0.7069	5	Price and Gestsdóttir (2006)	Seawater	0.7092	Åberg (1995)
Modern reindeer	0.7060	1	Åberg (1995)	Rainwater	0.7090	Åberg (1995)

The first published oxygen isotope ratios were measured from a small subset (*n* = 5) of the same Viking Age samples subjected to strontium isotope analysis by Price and Gestsdóttir (2006) (see Gestsdóttir & Price, 2006). The combined ^87^Sr/^86^Sr and δ^18^O values of these individuals indicate nonlocal provenance. Since then, Price and Gestsdóttir (2018) reported a mean δ^18^O_carbonate_ value of −4.86‰ ± 0.97 (range −6.9 to −2.2‰) measured from 117 individuals from pagan and early Christian Icelandic graves. The study suggests that Icelandic δ^18^O_carbonate_ values generally range between −7.0 and −4.0‰, with some individuals presenting with higher values (Price & Gestsdóttir, 2018). Additionally, bone and tooth samples of one early Settlement Period female migrant, *Bláklædda Konan* (LKS 1), were subjected to ^87^Sr/^86^Sr, δ^18^O_,_ δ^15^N, and δ^13^C isotope analyses, as well as lead (Pb) and strontium (Sr) trace element analyses (Montgomery & Jakob, 2015). The results of both studies provide a limited pool of comparative data. Modern precipitation of δ^18^O values in Iceland ranges from −13 to −8‰ (see Price et al., 2015, figure 20; Bowen, 2018) and in modern Icelandic groundwater from −8.8 to −8.2‰ (*n* = 11) (Friedrich & Schlosser, 2013).

### 2.3 Trace element analysis of humans in Iceland: Zinc (Zn), lead (Pb), barium (Ba), and strontium (Sr)

Assessing trace element variability between individuals within or at differing archaeological sites can indicate intrapopulation and interpopulation differences in geographic provenance, particularly when the elements are nonessential and not subject to homeostatic control. These applications are made possible because living organisms absorb elements through the consumption of water and food (Jaouen & Pons, 2017). No previous trace element analyses (i.e., Zn, Pb, Ba, and Sr) have been published on archaeological Icelandic human dental enamel samples prior to this study and thus there is no comparative contemporaneous context for this data. There is the possibility that results of such analyses can be used to aid in the assessment of geographic provenance of individuals excavated from Icelandic archaeological sites, particularly for those for which a link between human elemental levels and the geographical or cultural environment can be demonstrated (Burton, Price, Cahue, & Wright, 2003; Montgomery et al., 2014). These elements have been measured; however, in Icelandic geology, groundwater, soil, and plants (see Table 4). For example, Panek and Kepinska (2002) found no evidence for anthropogenic lead input in Icelandic soil and plants, particularly in comparison with the concentrations found in Sweden and Poland.

**Table 4:** Zinc, lead, and barium concentrations (ppm) found in two species of moss (*Racomitrium* sp. and *Drepanocladus* sp.), topsoils (andosols, regosols, leptosols, organic soil), bedrock (basalt), and groundwater in Iceland

Material	Zinc	Lead	Barium	Source
*Racomitrium sp*.	46.1	5.5	—	Panek and Kepinska (2002)
*Drepanocladus sp*.	54.1	5.9	—	Panek and Kepinska (2002)
Topsoil	83	5.8	—	Panek and Kepinska (2002)
Bedrock	63	4.7	75	Naimy (2008) and Panek and Kepinska (2002)
Groundwater	—	—	0.0036	Naimy (2008)

## 3 METHODS

In this study, bone samples from 50 humans (36 from Skriðuklaustur and 14 from Skeljastaðir) and 25 animals (from Skriðuklaustur) were subjected to isotope analysis for carbon (δ^13^C), nitrogen (δ^15^N) and sulfur (δ^34^S). The sample selection was informed by state of preservation, overall skeletal completeness, age, sex, and pathologies. Osteological and pathological analyses were conducted according to standard anthropological methods (e.g., Aufderheide & Rodriguez‐Martin, 2011; Brothwell, 1981; Buikstra & Ubelaker, 1994; Mitchell & Brickley, 2017; Ortner, 2003; Roberts & Connell, 2004; White, Black, & Folkens, 2011). The samples were selected from skeletal elements that did not present with pathological bone changes and were predominately taken from ribs, which record diet in the last few years of life. A previous study had undertaken dietary isotope analysis of 13 individuals from Skeljastaðir (Sveinbjörnsdóttir et al., 2010) and three of these individuals were reanalyzed here to control for interlaboratory differences. Unfortunately, no animal bones are preserved from Skeljastaðir. From Skriðuklaustur, bones samples representing a variety of animal species were sampled, including *Bos taurus sp*. (cattle), *Capra hircus sp*. or *Ovis aries sp*. (sheep or goat), *Equus* sp. (horse), *Canidae* sp. (dog or fox), *Phocidae* sp. (seal), *Cygnus cygnus* (swan), and marine fish, due to their differences in dietary resources and animal–human interactions. The bone collagen was extracted using a modified Longin method (Longin, 1971; O'Connell & Hedges, 1999) and the δ^13^C and δ^15^N stable isotope analyses were performed using a Costech elemental analyzer (ECS 4010) connected to a Thermo Delta V Advantage isotope ratio mass spectrometer. Finally, δ^34^S stable isotope analysis was performed using a Costech elemental analyzer (ECS 4010) connected to a Thermo Scientific Delta V Plus isotope ratio mass spectrometer (see Supplementary Materials). International standards, blanks and internal standards are routinely checked. Detailed analytical methods are provided in the Supplementary Materials.

Dental enamel samples from 31 of the same individuals sampled from Skriðuklaustur were also subjected to isotope analysis for strontium (^87^Sr/^86^Sr), oxygen (δ^18^O), and trace elements, including lead (Pb), strontium (Sr), zinc (Zn), and barium (Ba). Dental enamel samples were not selected from the remaining 19 individuals included in the bone collagen analysis due to various issues, including preservation, availability of material and ethical concerns over destructive analysis. The enamel samples were primarily selected from premolars, in which the enamel mineralizes within approximately 3 years at sometime between 2.5 and 8.5 years of age (AlQahtani, Hector, & Liversridge, 2010). In three individuals, only the third molars were available for sampling, the enamel of which mineralizes within about 4 years between 7.5 and 16.6 years of age (AlQahtani et al., 2010). Dental enamel samples were not sampled from Skeljastaðir because results from a previous study by Price and Gestsdóttir (2006) were available. The enamel samples were prepared following the procedure given in Montgomery (2002). All isotope and trace element concentrations were determined in the Department of Earth Sciences, Durham University. Carbon (δ^13^C) and oxygen (δ^18^O) isotope ratios were measured in the carbonate (CO_3_) component of tooth enamel by Thermo Fisher Scientific MAT 253 gas source mass spectrometer for isotope analysis. The ^87^Sr/^86^Sr ratios were determined using a Neptune multicollector inductively coupled plasma mass spectrometer (ICP‐MS). Finally, enamel samples were analyzed for Sr, Ba, Zn, and Pb by ICP‐MS (Thermo Scientific XSeries2) and the final enamel concentrations were then determined based on sample weights and total dilution volumes. Detailed analytical methods are provided in the Supplementary Materials.

## 4 RESULTS

### 4.1 Carbon, nitrogen, and sulfur isotope analysis in bone collagen

**Figure 2:**
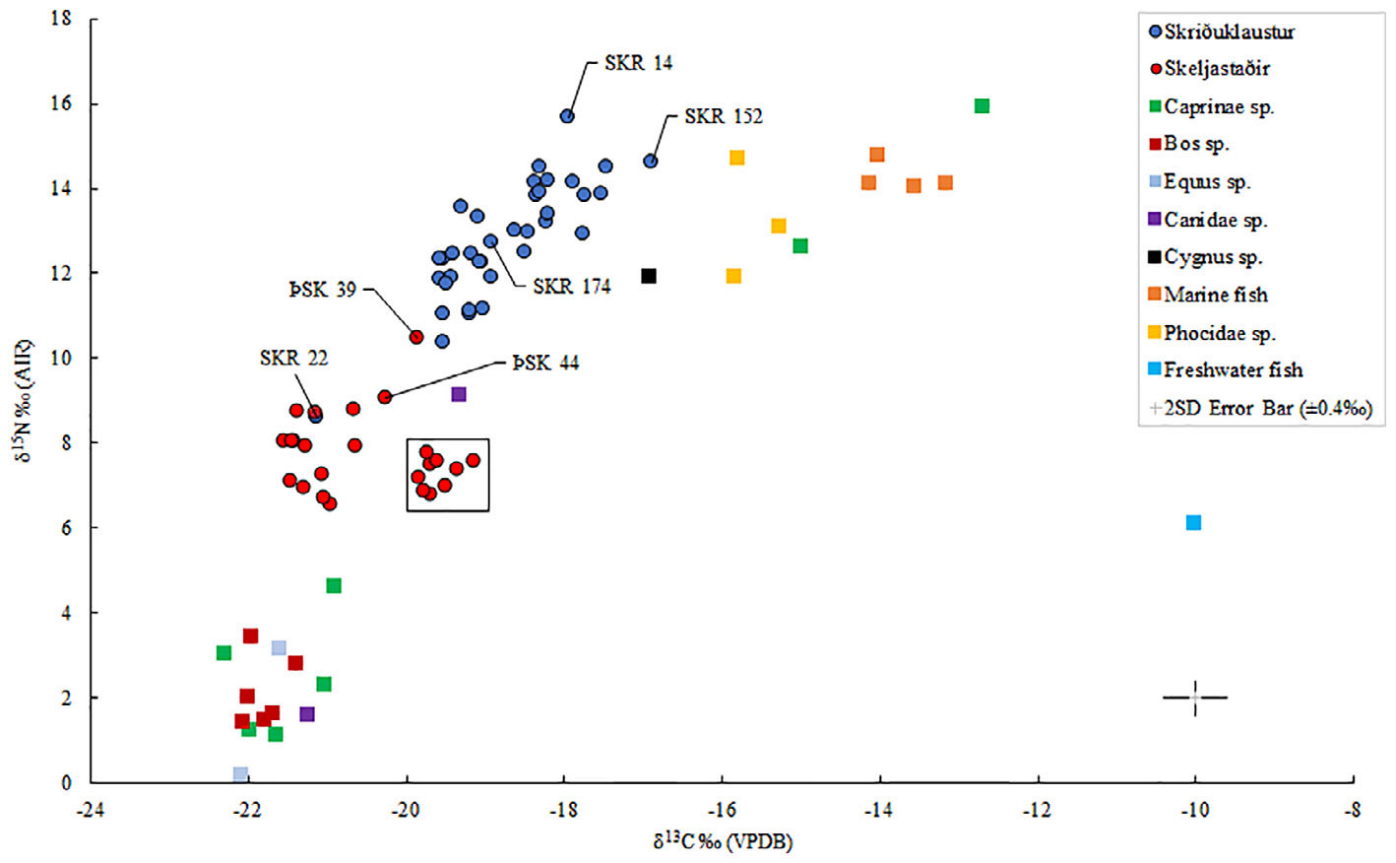
Plot of δ^13^C versus δ^15^N values for sampled individuals from Skriðuklaustur and Skeljastaðir. Also plotted are the δ^13^C versus δ^15^N values of the archaeological animal bone samples from Skriðuklaustur, except for freshwater fish, which were reported in Sayle et al. (2013). The data points within the black square and ÞSK 39 represent individuals from Skeljastaðir as reported in Sveinbjörnsdóttir et al. (2010). 2 *SD* error ± 0.4‰

All human and animal skeletal samples provided well preserved bone collagen with C:N atomic ratios (SKR C:N atomic mean of 3.2, ÞSK C:N atomic mean of 3.3) falling between 3.0 and 3.4 (see Ambrose, 1990; DeNiro 1985). No notable differences were observed between sex and age groups. However, two male individuals from Skriðuklaustur (SKR 135, lower, and SKR 172, higher) and one male from Skeljastaðir (ÞSK 44, higher) had outlying δ^34^S values. Descriptive statistics are presented in Table 5. Overall, at Skriðuklaustur (*n* = 36), the range and overall δ^13^C mean value indicate a marine dietary signal (closer to the marine dietary end member of −12.5‰) (see Table 5 and Figure 2), while at Skeljastaðir (see Table 5 and Figure 2) (*n* = 14), a terrestrial dietary signal (closer to the terrestrial dietary end member of −21‰) is indicated, based upon the values adopted by Arneborg et al. (1999) according to studies conducted on individuals from Norway, Canada, western Greenland and Sweden (see Sveinbjörnsdóttir et al., 2010). The δ^13^C and δ^15^N values determined by Sveinbjörnsdóttir et al. (2010) from Skeljastaðir are included in the means, bringing the total number of individuals to *n* = 24 (excluding δ^34^S values). The δ^13^C sample values and mean reported by Sveinbjörnsdóttir et al. (2010) are between 1 and 2‰ higher than those determined in this study. A δ^13^C offset of 1–2‰ was noted even among three samples that were reanalyzed during this research (ÞSK 16, 34 and 48) despite the very similar δ^15^N values reported in both studies. Therefore, the overall δ^13^C mean reported in the present analysis at Skeljastaðir is raised by approximately 1‰ when the two datasets are combined and calculated together. This offset in the stable isotope results is difficult to explain but may be due to differences in procedures in extraction methods, stable isotope analytical protocols, and instrumentation. Overall, the carbon and nitrogen isotope ratios in adult bone collagen from Skriðuklaustur show a diet dominated by mixed marine and C_3_ terrestrial protein resources, which is significantly different to the predominately C_3_ terrestrial protein diet seen at Skeljastaðir.

**Table 5:** Mean and *SD* for δ^13^C, δ^15^N, and δ^34^S analyses among sampled individuals from Skriðuklaustur and Skeljastaðir. At Skriðuklaustur, the δ^34^S means does not include SKR 174 due to an insufficient quantity of collagen for analysis, therefore for δ^34^S values *n* = 11 for males, *n* = 29 for adults, and *n* = 35 for all individuals. At Skeljastaðir, the δ^34^S means do not include the individuals sampled by Sveinbjörnsdóttir et al. (2010), therefore *n* = 8 for males, *n* = 6 for females, and *n* = 14 for all individuals in the determined δ^34^S values. The values for ÞSK 16, 34, and 48 from Sveinbjörnsdóttir et al. (2010) were not included in these means because they were also analyzed in this study

Skriðuklaustur					Skeljastaðir				
Sex	*n*	δ^13^C‰	δ^15^N‰	δ^34^S‰	Sex	*n*	δ^13^C‰	δ^15^N‰	δ^34^S‰
Males	12	−18.5 ± 0.8	13.0 ± 1.3	9.2 ± 2.0	Males	13	−20.4 ± 0.7	7.1 ± 2.1	9.4 ± 1.7
Females	18	−18.8 ± 0.7	12.6 ± 1.1	9.0 ± 1.4	Females	11	−20.6 ± 0.9	7.8 ± 0.9	9.4 ± 1.6
Nonadults	6	−19.0 ± 1.2	12.8 ± 2.5	8.6 ± 1.2	All individuals	24	−20.5 ± 0.8	7.8 ± 0.9	9.4 ± 1.6
All adults	30	−18.7 ± 0.7	12.7 ± 1.1	9.0 ± 1.7					
All individuals	36	−18.7 ± 0.8	12.8 ± 1.1	9.0 ± 1.6					

**Figure 3:**
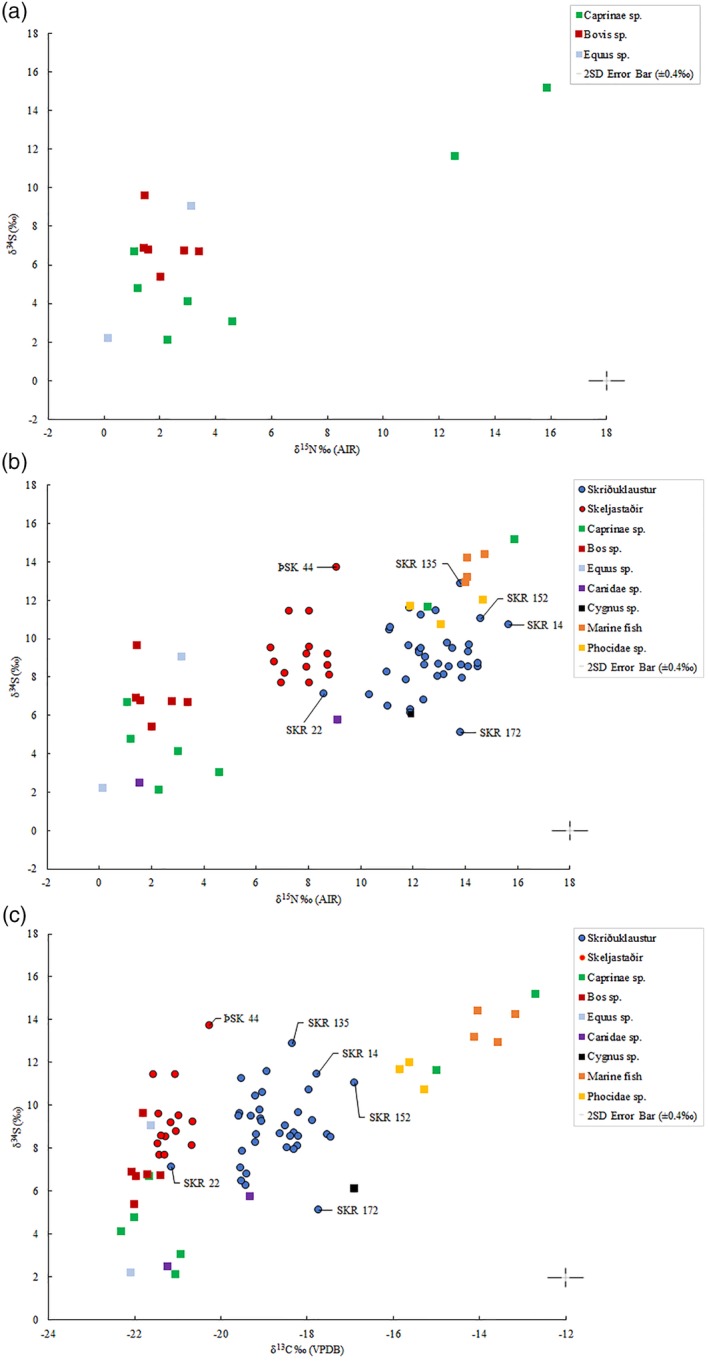
(a) Plot of δ^15^N versus δ^34^S values determined from the sampled herbivores from Skriðuklaustur. (b) Plot of δ^15^N versus δ^34^S values determined from the archaeological human and animal bone samples from both sites. (c) Plot of δ^13^C versus δ^34^S values determined from the archaeological human and animal bone samples from both sites. Notes: Freshwater fish (*n* = 12; not shown on plots) exhibit mean δ^13^C values of −9.8 ± 0.6‰, δ^15^N values of 5.9 ± 0.6‰, and δ^34^S values of −2.7 ± 1.4‰, according to Sayle et al. (2013). There was not enough collagen to measure δ^34^S for SKR 174

The mean values of δ^13^C, δ^15^N, and δ^34^S determined in animal bones (*n* = 25) from Skriðuklaustur are presented in Table 6 and plotted in Figures 1 and 2. The isotope values determined in the human bone samples can only be compared with animal baselines from Skriðuklaustur (Figure 2 and Table 6) and elsewhere in Iceland (see Table 2). The *Cygnus sp*. (swan) sample had relatively high δ^13^C (−16.9‰) and δ^15^N (11.9‰) values. While swans are generally herbivorous, they are known to incidentally consume the tiny animals (e.g., worms, fish, molluscs) that are often found in the weeds that they eat. The *Bos sp*. (cow) and *Equus sp*. (horse) samples showed δ^13^C and δ^15^N values consistent with herbivorous diets. Meanwhile, *Phocidae sp*. (seals) and saltwater fish samples had δ^13^C and δ^15^N values consistent with diets predominately derived from marine sources. Among *Canidae* species, one sample had a very low δ^15^N value of 1.6‰, while the other had a significantly higher δ^15^N value of 9.1‰. This difference may suggest that these two samples represent different species (e.g., domestic dog and wild arctic fox) or the same species with differing diets. Additionally, two *Caprinae sp*. (sheep/goat) samples, which must be domestic, displayed notably higher carbon and nitrogen isotope ratios (δ^13^C ~ −13.8‰, δ^15^N ~ 14.3‰, δ^34^S ~ 13.4‰) than the others (*n* = 5) (mean δ^13^C −21.6 ± 0.5‰, δ^15^N 2.5 ± 1.3‰, and δ^34^S 4.1 ± 1.6‰), indicating a diet substantially derived from marine resources (see Schulting et al., 2017) (see Figures 2 and 3a). For comparison, Sayle et al. (2013) reported sheep/goat samples with means of δ^13^C −21.2 ± 0.4‰, δ^15^N 2.5 ± 1.1‰, and δ^34^S 6.7 ± 1.9‰ but did not report any outliers in δ^15^N values. This provides evidence that notably different husbandry feeding practices were being followed for domestic animals.

**Table 6:** Ranges, mean values, and *SD* for δ^13^C, δ^15^N, and δ^34^S analyses among sampled animal bones from Skriðuklaustur. The freshwater fish range and mean were taken from Sayle et al. (2013). *Equus* sp., *Phocidae* sp., and *Canidae* sp. were only represented by two samples each so a true mean has not been provided. *Cygnus* sp. is only represented by one sample with isotope values reported under the mean columns. For comparison, see Table 2

Common name	Species	*n*	Range δ^13^C‰	Mean δ^13^C‰	Range δ^15^N‰	Mean δ^15^N‰	Range δ^34^S‰	Mean δ^34^S‰
Goat/sheep	*Caprinae sp*.	7	−22.3 to −12.7	−19.4 ± 0.9	1.1–15.9	5.8 ± 5.5	2.1 to 15.2	6.8 ± 4.5
Cow	*Bos sp*.	6	−22.1 to −21.4	−21.8 ± 0.2	1.4–3.4	2.1 ± 0.7	5.4 to 9.6	7.0 ± 1.3
Horse	*Equus sp*.	2	−22.1 to −21.6	~−21.8	0.2–3.2	~1.7	2.2–9.0	~5.6
Dog or fox	*Canidae sp*.	2	−21.2 to −19.3	~−20.3	1.6 to 9.1	~ 5.4	2.5–5.7	~4.1
Swan	*Cygnus sp*.	1	—	−16.9	—	11.91	—	6.1
Seal	*Phocidae sp*.	3	−15.8 to −15.3	−15.6 ± 0.2	11.9–14.7	13.2 ± 1.1	10.7 to 12.0	11.4 ± 0.5
Freshwater fish	—	12	−11.4 to −9.1	−9.8 ± 0.6	5.0–6.8	5.9 ± 0.6	−4.2 to −0.2	−2.7 ± 1.4
Saltwater fish	—	4	−14.0 to −13.2	−13.7 ± 0.4	14.0–14.8	14.3 ± 0.3	12.9–14.4	13.7 ± 0.6
Herbivores	—	15	−22.3 to −12.7	−20.7 ± 2.7	0.17–15.9	3.8 ± 4.3	2.1–15.2	6.9 ± 3.5

### 4.2 Strontium, oxygen, and carbon isotope and trace element analysis in dental enamel

The δ^18^O, δ^13^C, and ^87^Sr/^86^Sr (*n* = 31) determined from the dental enamel samples are presented in Table 7 and isotope ratio means and ranges are presented in Table 8. The humans from Skriðuklaustur range from 0.7060 to 0.7088 and all fall between the lower geological end member of the basalt and the upper end member for the Icelandic biosphere of rain and seawater, that is, 0.7030–0.7092. All the humans and animals are therefore consistent with origins in Iceland or other regions of basaltic or marine limestones, the only two types of rocks which are known to produce biosphere values below 0.7092. The human δ^18^O_carbonate_ values range from 22.8 to 25.0‰, mean = 24.2 ± 0.6‰. Using the equations provided in Chenery, Pashley, Lamb, Sloane, and Evans (2012) (δ^18^O_phosphate_ = 1.0322 × δ^18^O_carbonate_ − 9.6849 and δ^18^O_drinkingwater_ = 1.590 × δ^18^O_carbonate[VSMOW]_ − 48.634), this equates to a δ^18^O_phosphate_ range of 13.9–16.1‰ and mean of 15.3 ± 0.6‰. The δ^18^O_drinkingwater_ values range from −12.3 to −8.9‰. Converting enamel δ^18^O to precipitation significantly increases the uncertainty on individual measurements, which according to Chenery et al. (2012) is ±1‰ (2 *SD*). Nonetheless, such a range for human enamel fits within the annual δ^18^O range for modern precipitation in Iceland (−13 to −8‰) (see Price et al., 2015, figure 20; Bowen, 2018) and is close to values obtained from modern groundwater (range −8.8 to −8.2‰; *n* = 11) (Friedrich & Schlosser, 2013). It is possible that changes in past climate such as the Little Ice Age may have an impact on the values of contemporaneous drinking water sources in the North Atlantic region (Daux, Lécuyer, Adam, Martineau, & Vimeux, 2005; Fricke, O'Neil, & Lynnerup, 1995) but these results define a δ^18^O_dw_ range in excess of 3‰ at −12.3 to −8.9 ± 1‰ (2 *SD*) for medieval humans who appear to be of Icelandic origin with regards to their strontium isotope ratios. The δ^13^C_carbonate_ values, which derive from whole diet during childhood, range from −16.6 to −12.9‰ with a mean of −15.2 ± 0.8‰. All individuals, aside from SKR 10 and SKR 14, fell within the expected range of a diet primarily derived from C_3_ terrestrial resources (−17.0 to −14.0‰; see Kellner & Schoeninger, 2007; Froehle, Kellner, & Schoeninger, 2012; Neil, Montgomery, Evans, Cook, & Scarre, 2017).

**Table 7:** All isotope and trace element data from the Skriðuklaustur and Skeljastaðir samples. Samples labeled with italics indicate δ^13^C and δ^15^N results reported by Sveinbjörnsdóttir et al. (2010). All strontium concentrations from Skeljastaðir (ÞSK), conducted on first molars unless unavailable, indicate results reported by Gestsdóttir and Price (2003, 2006) and Price and Gestsdóttir (2006). Enamel samples from SKR 30, 130, 144, 146, and 163 were unavailable or not preserved. δ^34^S is not reported for SKR 174 due to insufficient collagen for analysis

Sample	Tooth	Bone	Sex	Age	^87^Sr/^86^Sr	δ^18^O_p_‰	δ^18^O_dw_‰	δ^13^C_carb_‰	δ^13^C_co_‰	δ^15^N_co_‰	δ^34^S_co_‰	Sr ppm	Pb ppm	Zn ppm	Ba ppm
SKR 4	LPM2 max	Rib	M	OA	0.70706	13.9	−12.3	−15.4	−19.6	11.9	9.5	47.84	9.60	115.94	0.27
SKR 10	RM3 max	Rib	F	OA	0.70874	14.4	−11.5	−13.2	−19.2	11.0	8.26	156.68	0.30	145.83	0.51
SKR 14	RPM1 mand	Rib	—	NA	0.70887	15.6	−9.7	−12.9	−17.9	15.7	10.72	140.41	0.58	119.40	0.25
SKR 22	LPM1 mand	Rib	—	NA	0.70746	15.7	−9.5	−15.3	−21.1	8.6	7.10	46.04	2.65	112.90	0.11
SKR 23	RPM2 mand	Rib	F	YA	0.70798	15.8	−9.4	−15.3	−19.2	11.1	10.43	72.39	4.08	90.91	0.80
SKR 29	RPM1 mand	Rib	F	YA	0.70753	14.7	−11.1	−15.5	−19.2	12.5	8.63	40.80	2.28	73.51	0.41
SKR 30	—	Rib	F	OA	—	—	—	—	−19.5	12.3	11.23	—	—	—	—
SKR 33	LPM2 mand	Rib	F	OA	0.70859	15.9	−9.3	−14.7	−18.2	14.2	9.64	97.45	1.69	90.85	0.43
SKR 46	LPM2 mand	Rib	—	NA	0.70845	15.9	−9.3	−14.8	−19.1	12.3	9.38	88.18	2.73	101.84	0.17
SKR 65	LPM2 max	Rib	F	YA	0.70742	13.8	−12.5	−16.4	−19.1	13.3	9.75	44.14	3.51	66.99	0.81
SKR 81	LPM2 mand	Rib	F	YA	0.70757	16.0	−9.1	−15.7	−19.4	11.9	6.28	45.15	0.73	70.79	0.69
SKR 91	LPM2 mand	Rib	M	YA	0.70790	15.6	−9.7	−15.1	−19.1	12.3	9.26	52.90	0.53	91.57	0.17
SKR 100	LPM2 mand	Rib	M	YA	0.70834	15.2	−10.3	−14.5	−18.9	11.9	11.58	93.70	2.68	104.60	0.40
SKR 115	LPM1 mand	Rib	M	OA	0.70686	15.9	−9.3	−15.7	−18.2	13.2	8.11	71.20	0.35	95.30	0.60
SKR 122	LPM2 max	Parietal	F	YA	0.70747	14.4	−11.6	−15.5	−19.3	13.5	9.49	44.40	1.38	134.59	0.62
SKR 126	LPM2 max	Rib	F	OA	0.70781	15.6	−9.7	−15.2	−18.6	13.0	8.67	67.25	0.51	126.08	0.63
SKR 128	LPM2 max	Rib	F	OA	0.70813	14.9	−10.8	−16.0	−17.5	13.9	8.61	64.13	0.51	87.03	0.29
SKR 130	—	Rib	M	OA	—	—	—	—	−17.5	14.5	8.52	—	—	—	—
SKR 135	LM3 max	Rib	M	YA	0.70727	16.1	−8.9	−15.2	−18.3	13.8	12.86	45.83	0.64	73.10	0.72
SKR 144	—	Rib	F	YA	—	—	—	—	−18.5	13.0	8.01	—	—	—	—
SKR 146	—	Rib	—	NA	—	—	—	—	−18.3	14.5	8.70	—	—	—	—
SKR 150	RPM2 mand	Rib	M	YA	0.70726	14.4	−11.5	−16.1	−18.4	14.2	8.54	43.69	0.39	47.30	0.46
SKR 152	LPM2 mand	Rib	M	YA	0.70832	15.6	−9.7	−14.5	−16.9	14.6	11.06	53.13	0.23	112.00	0.27
SKR 155	LM3 max	Rib	M	OA	0.70840	15.4	−10.0	−14.5	−18.5	12.5	9.05	73.54	0.21	123.89	0.29
SKR 163	—	Rib	—	NA	—	—	—	—	−18.3	13.9	7.95	—	—	—	—
SKR 167	RPM1 mand	Rib	M	YA	0.70723	14.4	−11.5	−16.6	−19.5	10.4	7.08	18.31	0.26	67.54	0.17
SKR 169	RPM1 max	Rib	F	OA	0.70809	15.5	−9.9	−14.9	−19.0	11.2	10.57	82.28	0.33	70.56	0.70
SKR 172	RPM1 max	Rib	M	OA	0.70684	15.2	−10.4	−15.1	−17.7	13.8	5.10	43.18	0.34	81.92	0.13
SKR 174	RC max	Femur	M	OA	0.70602	16.0	−9.1	−15.5	−18.9	12.7	—	74.97	1.46	104.97	0.61
SKR 181	LPM2 max	Temporal	F	OA	0.70857	16.0	−9.1	−14.5	−19.4	12.4	6.81	79.55	0.41	116.02	0.18
SKR 189	LPM1 mand	Rib	F	YA	0.70700	15.1	−10.4	−15.8	−19.6	12.3	9.47	43.90	9.40	145.50	0.40
SKR 195	LPM2 mand	Rib	F	YA	0.70874	15.6	−9.7	−14.0	−17.9	14.1	9.28	111.26	0.45	43.76	0.51
SKR 201	LPM1 max	Rib	F	YA	0.70816	15.1	−10.4	−16.3	−18.2	13.4	8.54	80.90	1.36	141.54	0.23
SKR 221	RPM2 mand	Rib	—	NA	0.70767	15.5	−9.8	−15.3	−19.5	11.8	7.84	25.79	0.13	98.61	0.22
SKR 226	RPM1 mand	Rib	F	OA	0.70778	15.4	−10.1	−15.1	−19.5	11.1	6.48	87.33	0.61	75.60	0.46
SKR 241	LPM1 mand	Rib	F	OA	0.70800	15.3	−10.1	−15.4	−17.8	12.9	11.43	50.39	3.12	88.33	0.30
ÞSK 2	?	?	F	OA	0.70905	—	—	—	−19.7	7.5	—	—	—	—	—
ÞSK 3	—	Rib	F	OA	—	—	—	—	−21.0	6.6	8.5	—	—	—	—
ÞSK 4	—	Rib	F	YA	—	—	—	—	−20.7	8.8	7.7	—	—	—	—
ÞSK 5	?	?	F	YA	0.70591	—	—	—	−19.8	7.2	—	—	—	—	—
ÞSK 12	?	?	F	OA	0.70562	—	—	—	−19.2	7.6	—	—	—	—	—
ÞSK 15	?	?	F	OA	0.70615	—	—	—	−19.6	7.6	—	—	—	—	—
ÞSK 16	?	Rib	F	OA	0.70754	—	—	—	−21.0	6.7	11.5	—	—	—	—
ÞSK 17	?	Rib	F	OA	0.70662	—	—	—	−21.2	8.7	9.6	—	—	—	—
ÞSK 26	?	?	M	OA	0.70610	—	—	—	−19.7	6.8	—	—	—	—	—
ÞSK 29	—	Rib	M	OA	—	—	—	—	−21.5	7.1	9.5	—	—	—	—
ÞSK 32	?	Rib	M	YA	0.70685	—	—	—	−21.4	8.8	8.1	—	—	—	—
ÞSK 34	—	Rib	M	YA	—	—	—	—	−20.3	9.1	8.8	—	—	—	—
ÞSK 37	?	Rib	M	OA	0.70900	—	—	—	−20.6	7.9	9.1	—	—	—	—
ÞSK 38	?	?	M	OA	0.70608	—	—	—	−19.5	7.0	—	—	—	—	—
ÞSK 39	?	?	F	OA	0.70972	—	—	—	−19.9	10.5	—	—	—	—	—
ÞSK 41a	?	?	M	YA	0.70704	—	—	—	−19.7	7.8	—	—	—	—	—
ÞSK 41b	—	Rib	M	OA	—	—	—	—	−21.3	7.9	8.2	—	—	—	—
ÞSK 42	?	Rib	M	OA	0.70653	—	—	—	−21.4	8.0	8.6	—	—	—	—
ÞSK 44	—	Fibula	M	OA	—	—	—	—	−21.6	8.1	13.7	—	—	—	—
ÞSK 47	?	?	M	YA	0.70682	—	—	—	−19.8	6.9	—	—	—	—	—
ÞSK 48	?	Rib	M	OA	0.70614	—	—	—	−21.4	8.0	9.2	—	—	—	—
ÞSK 51	—	Rib	F	OA	—	—	—	—	−21.1	7.3	11.4	—	—	—	—
ÞSK 54	—	Rib	F	YA	—	—	—	—	−21.3	7.0	7.7	—	—	—	—
ÞSK 60	?	?	M	OA	0.70718	—	—	—	−19.4	7.4	—	—	—	—	—


Abbreviations: δ^18^O_p_, δ^18^O_phosphate VSMOW_ (‰); δ^18^O_dw_, δ^18^O_drinkingwater VSMOW_ (‰); δ^13^C_carb_, δ^13^C_carbonate VPDB_ (‰); δ^13^C_co_, δ^13^C_collagen_ (‰); δ^15^N_co_, δ^15^N_collagen_ (‰); δ^34^S_co_, δ^34^S_collagen_ (‰); ÞSK, Skeljastaðir; F, female; LPM1, left first premolar; LPM2, left second premolar; LM3, left third molar; M, male; mand, mandible; max, maxilla; NA, nonadult (<17); OA, older adult (36+); RC, right canine, RPM1, right first premolar; RPM2, right second premolar; RM3, right third molar; SKR, Skriðuklaustur; Sr/Pb/Zn/Ba ppm, strontium/lead/zinc/barium parts per million; YA, young adult (17–36).

**Table 8:** Medians and ranges of trace elements and means and ranges of isotope ratios determined in the human dental enamel samples from Skriðuklaustur. Samples SKR 100, 115, and 189 were run during the second analytical session which gave the average ^87^Sr/^86^Sr value and reproducibility for the international isotope reference material NBS987 as 0.710266 ± 0.000009 (2 *SD*; *n* = 6) while all others were run in the first analytical session which gave 0.710258 ± 0.000013 (2 *SD*; *n* = 12)

Samples	*n*	Lead (Pb) ppm	Barium (Ba) ppm	Zinc (Zn) ppm	Strontium (Sr) ppm
Male	11	0.39	0.29	95.3	52.9
Female	16	1.05	0.49	89.59	69.82
Nonadult	4	1.62	0.20	107.37	67.11
All	31	0.61	0.40	95.3	64.13
Ranges (all)	31	0.13–9.40 ppm	0.11–0.81 ppm	43.76–145.83 ppm	25.79–156.68 ppm

Samples	*n*	^87^Sr/^86^Sr	δ^18^O_phosphate‰ (VSMOW)_	δ^18^O_drinkingwater‰ (VSMOW)_	δ^13^C_carbonate‰ (VPDB)_
Male	11	0.70741 ± 0.00144 (2 *SD*)	15.2 ± 0.7	−10.2 ± 1.1	−15.3 ± 0.6
Female	16	0.70797 ± 0.00099 (2 *SD*)	15.2 ± 0.6	−10.3 ± 1.0	−15.2 ± 0.8
Nonadult	4	0.70811 ± 0.00114 (2 *SD*)	15.7 ± 0.1	−9.6 ± 0.2	−14.6 ± 1.0
All	31	0.70779 ± 0.00132 (2 *SD*)	15.3 ± 0.6	−10.2 ± 1.0	−15.2 ± 0.8
Ranges (all)	31	0.70602–0.70887	13.8–16.1	−12.3 to −8.3	−16.6 to −12.9

The trace element (Ba, Zn, Pb, and Sr) concentrations (*n* = 31) determined from the dental enamel samples are presented in Table 7 and Supplementary Figures S1 and S2 and the medians and ranges in Table 8. Trace element bioavailability is affected by the complex interaction of numerous variables that occur during mineral metabolism. Strontium is a nonessential trace element that enters biological tissues via calcium pathways following the ingestion and metabolization of food and drink. In omnivores, such as humans, known antagonisms, and synergisms with other elements and food types result in skeletal strontium being predominately derived from the plant part of the diet. It passively enters and remains in the skeleton by substituting for calcium and is not subject to homeostatic control. The amount of strontium incorporated into skeletal tissues is dependent on dosage but is thought to reflect the amount of strontium that is bioavailable from the local environment, diet, dietary calcium, and any strontium released from the skeleton through calcium homeostasis (Montgomery, Evans, Chenery, Pashley, & Killgrove, 2010). Similarly, barium is a sensitive dietary indicator that, like strontium, is not subject to homeostatic control but is absorbed by plants in smaller amounts and may be discriminated according to rising trophic chain position (Szostek, Głąb, & Pudło, 2009). In this study, the strontium concentrations range from 25.8 to 156.7 ppm with a median of 64.1 ppm and the barium concentrations determined in the same individuals range from 0.11 to 0.81 ppm with a median of 0.40 ppm. Ancient and modern dental enamel concentrations of zinc reportedly range from 58 to 2,100 ppm according to a combination of studies reviewed by Ezzo (1994). Some studies have identified a positive correlation between zinc and lead concentrations and areas of increased urbanization and industrialization (see Ezzo, 1994; Tvinnereim, Eide, Riise, Fosse, & Wesenberg, 1999). Lead concentrations in dental enamel as associated with geological or environmental exposure, rather than from anthropogenic exposure, are generally less than ~0.7 ppm (Millard et al., 2014; Montgomery et al., 2010). The zinc concentrations in this study range from 43.8 to 145.8 ppm with a median of 95.3 ppm, while the lead concentrations range from 0.13 to 9.4 ppm with a median of 0.61 ppm.

## 5 DISCUSSION

### 5.1 Monasteries, travel, and trade

Medieval Icelandic monasteries were normally located on major travel routes or near coastal settlements where most of the population resided at the time. Although Skriðuklaustur now appears to be situated in a remote inland valley, during its occupation it was centrally located on a major routeway between the northern and southern parts of the country (Kristjánsdóttir, 2012, p. 296; Kristjánsdóttir, 2016). Until the 17th century, when the route closed due to climate change, pilgrims, patients, fish traders and other individuals easily traveled over the Vatnajökull glacier to reach Skriðuklaustur in the Fljótsdalur valley (Björnsson, 2009, p. 243; Kristjánsdóttir, 2016) (Figure 1). Severe environmental and epidemiological conditions were likely to have been major catalysts for the movement of people to Skriðuklaustur, which represents one of the largest skeletal assemblages excavated in Iceland and a high prevalence of pathological conditions. The Black Death first came to Iceland at the beginning of the 15th century, killing more than half of the population (Karlsson, 2000, pp. 114–117; Kristjánsdóttir, 2016; Júlíusson, 2018). The second wave of the Plague occurred around AD 1495–1496, just after the establishment of monastery (Kristjánsdóttir, 2016). Furthermore, the only confidently diagnosed cases of treponemal disease in Iceland were found at Skriðuklaustur (Kristjánsdóttir, 2012; Walser, Kristjánsdóttir, Gowland, & Desnica, 2018), indicating that immediately following the plague, an outbreak of treponemal disease occurred in Iceland contemporaneously with the European epidemic of the late 15th century (see Walker, Power, Connell, & Redfern, 2014). The Black Death and other plagues in Iceland also coincided with climate changes such as sustained summer rains and cooling weather, which lead to grass and crop failure, increased disease burden, caused food shortages and increased the number of homeless people (Kristjánsdóttir, 2016).

It is known that the brethren residing at Skriðuklaustur aimed to buy farms near the coast: access to the valuable resources (e.g., fish, driftwood, whales, and seals) that coastal sites offered being the probable driver. Those running monasteries found a variety of ways for earning money, including participation in local and international trade (Steinsson, 1965, p. 108; Steinsson, 1966; Kristjánsdóttir, 2016). For example, refined sulfur, an important commodity in medieval trade was found at Skriðuklaustur (Kristjánsdóttir, 2012; Mehler, 2011), possibly for medicinal uses or the production of vermilion (Mehler, 2015). Other notable imports include an effigy of Saint Barbara, a monastic trumpet and rare ceramic pottery imported from France (Kristjánsdóttir, 2012; Mehler, Kristjánsdóttir, & Kluttig‐Altmann, 2018). Other sources of income included donations from benefactors from the local community, the sale of books and payment for medicinal treatment and community charity (Kristjánsdóttir, 2016). The Skriðuklaustur monastery also partly depended upon foreign commerce for dietary resources. A large amount of fish bones, primarily from cod (*Gadus morhua*), ling (*Molva molva*), haddock (*Melanogranmus aeglefinus*), shark, and rays were also found during the excavation at Skriðuklaustur, indicating that marine fish were indeed an important dietary component. Smaller fish (60–80 cm in length) are generally found in the Greenland Sea and around the northern and eastern coasts of Iceland, while larger fish (often over 100 cm), such as those found at Skriðuklaustur, are normally caught around the southern and western coasts (Kristjánsdóttir, 2016). It is important to note that larger fish tend to reach higher trophic levels and elevated isotope values (Schoeninger & DeNiro, 1984; Häberle et al., 2016). Fish were both dietarily and culturally important at Skriðuklaustur, where religious fasting was practiced (Kristjánsdóttir, 2017). Zooarchaeological research demonstrated that fresh fish were regularly consumed at the monastery unlike at most inland sites where only dried fish are normally found (Hamilton‐Dyer, 2010; Pálsdóttir, 2006). This notable difference was undoubtedly connected with the religious fasting practiced at the monastery (Pálsdóttir, 2006). However, bipedal animals such as poultry were also permitted for consumption during fasting periods among some Augustinian and other monastic orders (Kristjánsdóttir, 2017) and seals were also still consumed during the fast.

**Figure 4:**
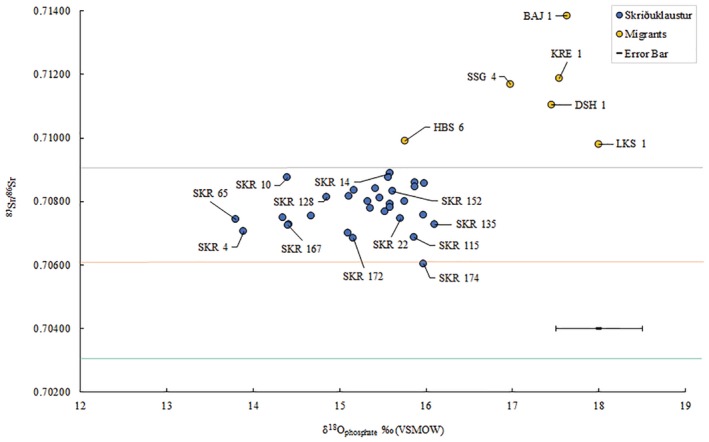
A plot of δ^18^O_phosphate_ and ^87^Sr/^86^Sr values for the enamel samples from individuals buried at Skriðuklaustur (dark blue). For comparison, Settlement period migrants (Gestsdóttir & Price, 2006), including LKS 1 (*Bláklædda konan*) (Montgomery & Jakob, 2015), are indicated by the yellow points. Icelandic bedrock (0.7030–0.7037), sheep (0.7059–0.7069), and seawater (0.7092) are indicated by the horizontal lines (Sigmarsson et al., 1992; Price & Gestsdóttir, 2006; also see Table 3). Error bar for 2 *SD* for δ^18^O at ±0.5‰ and ^87^Sr/^86^Sr at 0.002

The results of bone collagen stable isotope analyses for carbon and nitrogen on individuals from Skriðuklaustur align with the historical and zooarchaeological evidence, demonstrating a diet substantially derived from marine and potentially freshwater fish protein in some individuals (see Figure 2). No sex‐based differences in dietary intake were noted. Based on a trophic shift of +1‰ for carbon and 5.5 ± 0.5‰ for nitrogen (see Fernandes, 2015; Sayle et al., 2016) resulting in a δ^13^C value of −20.7‰ and a δ^15^N value of 7.7 ± 0.5‰, none of the individuals at Skriðuklaustur subsisted on an entirely terrestrial protein diet in adulthood. However, if the two sheep that grazed on seaweed and/or fishmeal (see Figure 2) are included, then a + 5.5 ± 0.5‰ trophic shift results in a δ^15^N value of 9.2 ± 0.5‰. In this case, one individual (SKR 22; 16.5–18.5 years old) with a significantly lower δ^15^N value (8.6‰) consumed a solely terrestrial diet. Since the individual (SKR 22) exhibits a cleft lip (cleft premaxilla) and palate (cleft maxilla) (Barnes, 2012) (Figure 4) and a differential diagnosis of treponemal disease based on gummatous cranial and tibial lesions (see Aufderheide & Rodriguez‐Martin, 2011; Hackett, 1976; Ortner, 2003), poor overall health and dietary restrictions are suggested. Palatal clefts or perforations can occur due to several reasons, including late stage syphilis, and often cause significant difficulties in eating, drinking and speaking (Ilczuk‐Rypuła, Pietraszewska, Kempa, Zalewska‐Ziob, & Wiczkowski, 2017; Patil, 2016). Additionally, individuals born with cleft palate generally have difficulties breastfeeding and take longer to adapt to eating solid foods than other children (Müldner et al., 2009; Wiet, Biavati, & Rocha‐Worley, 2017). Similarly, Müldner et al. (2009) noted a high‐status individual with cleft palate from Whithorn Cathedral priory, Scotland (13th–14th century) that also exhibited δ^13^C and δ^15^N values consistent with a predominately terrestrial diet, unlike the mixed marine and terrestrial diet shown by the other bishops and clerics buried at the priory. SKR 22 contrasts with SKR 14, another nonadult who appears to have been one of the highest marine protein consumers throughout life.

### 5.2 Mobility and geographic provenance

**Figure 5:**
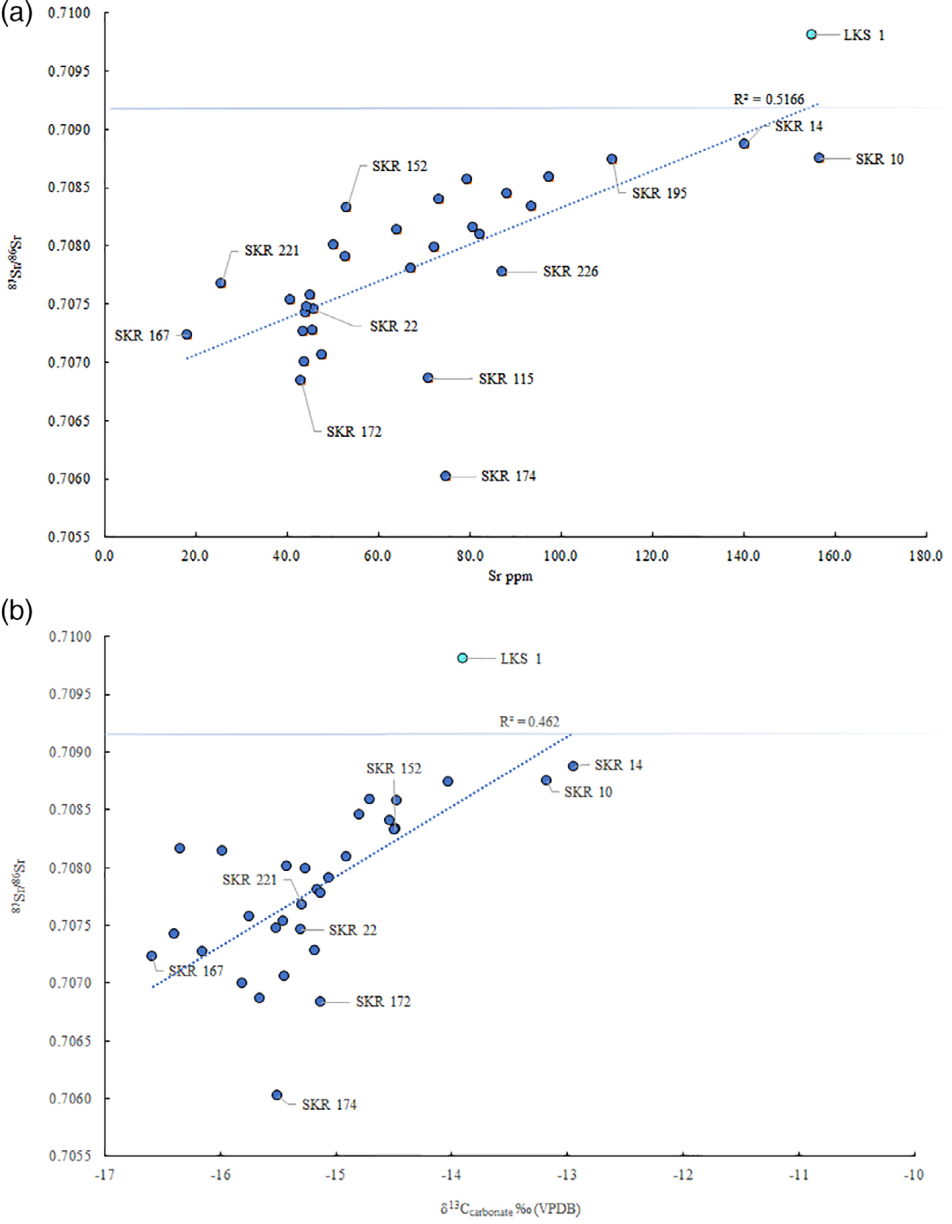
(a) Plot of ^87^Sr/^86^Sr and concentration (ppm) from the dental enamel samples from individuals buried at Skriðuklaustur. The teal marker labeled LKS 1 represents an early Settlement Period migrant to Iceland known as *Bláklædda konan* (Montgomery & Jakob, 2015). The horizontal line marks the ^87^Sr/^86^Sr value of seawater (0.7092). Excluding SKR 174, the *R* ^2^ value is .67 and if both SKR 174 and LKS 1 are excluded the *R* ^2^ value is 0.58. (b) Plot of ^87^Sr/^86^Sr with δ^13^C_carbonate_ values determined from dental enamel samples from Skriðuklaustur. Excluding SKR 174, the *R* ^2^ value is 0.52 and if both SKR 174 and LKS 1 are excluded the *R* ^2^ value is 0.50

**Figure 6:**
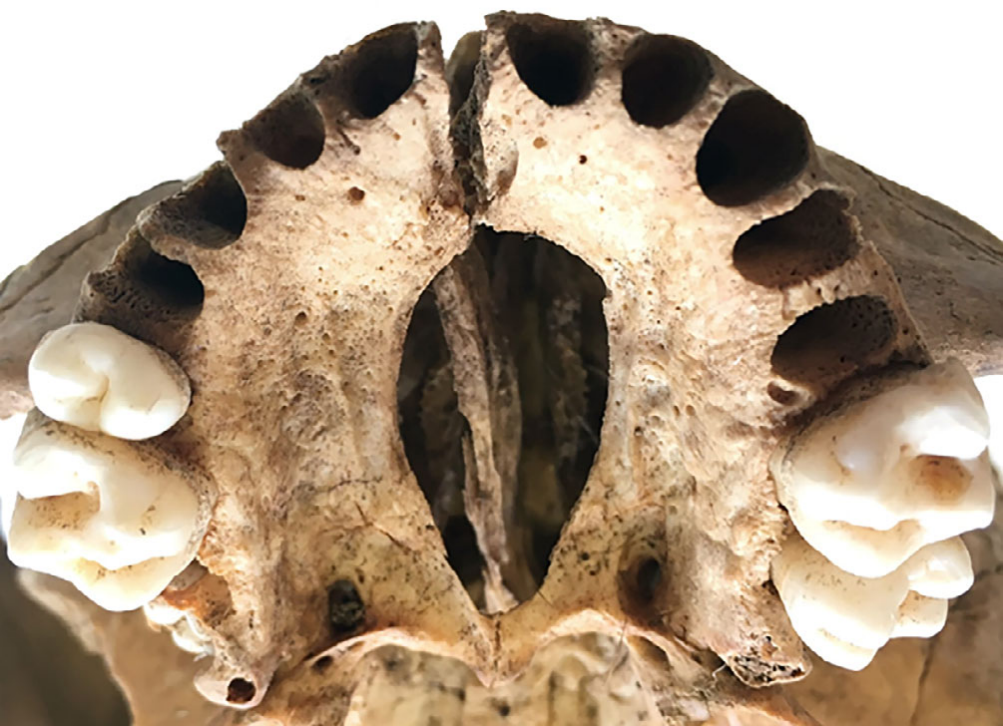
Cleft maxilla and premaxilla from SKR 22, inferior view. © Joe W. Walser III / National Museum of Iceland

Prior to this research, the origin of the individuals buried at Skriðuklaustur was unknown. Due to the site's hospital, monastic, and trade network functions, it was hypothesized that the individuals buried there could be represented by locals, foreign traders, pilgrims or patients seeking treatment from elsewhere in the country or from abroad (Kristjánsdóttir, 2012; Kristjánsdóttir, 2017). The strontium and oxygen isotope results demonstrate that all the individuals sampled during this study were likely to be of indigenous origin (see Table 7 and Figure 5). None of the individuals exceed the ^87^Sr/^86^Sr value of rain and seawater (0.7092), which implies an Icelandic origin. The possibility that some individuals had originally resided on chalk, which has a range of 0.708–0.709, (e.g., Denmark and Southern Britain) cannot be completely ruled out (see Evans, Chenery, & Montgomery, 2012; Evans, Montgomery, Wildman, & Boulton, 2010; Montgomery et al., 2014). However, the δ^18^O_phosphate_ values are inconsistent with an origin in Britain/Ireland, where δ^18^O_phosphate_ would have to fall within the range of 16.3–19.1‰ (see Montgomery et al., 2014). Furthermore, the δ^18^O_phosphate_ range is ~2‰, which is normal for a single temporally contemporaneous population, indicating that these individuals most likely represent a local group of people. Overall, the results suggest that the individuals sampled from the temporally constrained (1493–1554 AD) cemetery at Skriðuklaustur were of Icelandic origin. As the ^87^Sr/^86^Sr value approaches the value of rain and/or seawater, δ^13^C_carbonate_ values move away from a wholly terrestrial value thereby suggesting a higher input of marine‐derived strontium and carbon into the diet (Figure 6). However, it is important to note that δ^13^C_carbonate_ reflects whole diet (e.g., fat, carbohydrates, and protein) rather than just the protein component of the diet. Nonetheless, the variability in the plot of δ^13^C_carbonate_ and ^87^Sr/^86^Sr may indicate that some individuals (e.g., SKR 167) consumed a wholly terrestrial diet while living further from the coast (lower δ^13^C_carbonate_, more basaltic‐derived ^87^Sr/^86^Sr values) during childhood, while others with higher δ^13^C_carbonate_ and more marine‐derived ^87^Sr/^86^Sr values (e.g., SKR 10 and 14) appear to have consumed marine resources and lived near the coast where sea spray and splash were more prevalent. This positive correlation is strengthened by the Sr concentrations which are also positively correlated with δ^13^C_carbonate_ and ^87^Sr/^86^Sr: as Sr isotope ratios approach the seawater value the amount of Sr in the enamel increases (see Figure 6). This suggests that the higher values are indicative of individuals that grew up in a coastal area where the food web was impacted by marine sea spray and splash, consumption of seaweed or seaweed‐eating fauna, while those with lower ratios and concentrations resided further inland where the basalt dominated the food chain. The correlation between these three parameters is logical but rarely observed so clearly in human populations—highlighting the advantages of studying populations inhabiting the geologically homogenous islands providing two‐end member systems—and suggests that it is possible to use isotope analysis to identify residence within different residential zones in Iceland.

**Figure 7:**
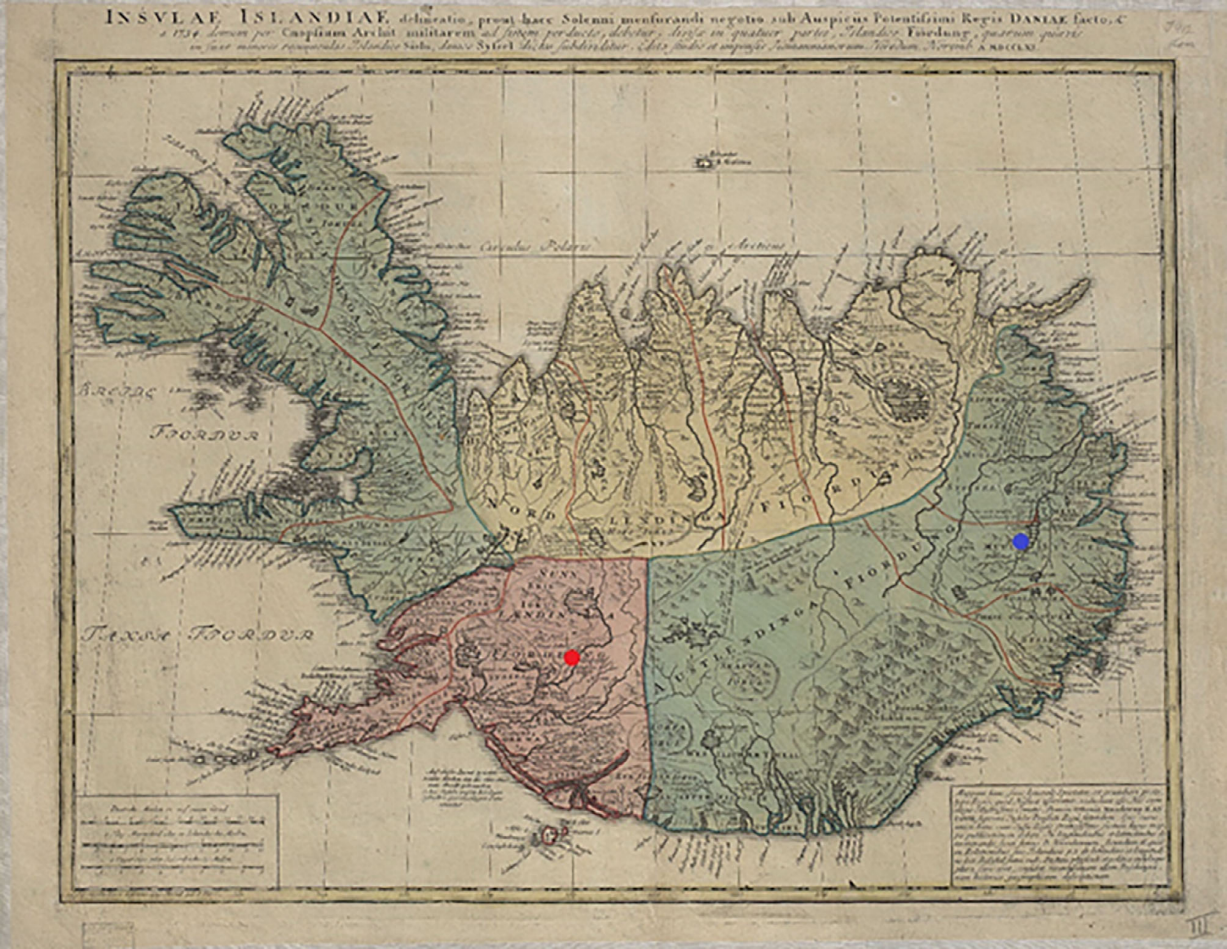
A medieval map of Iceland depicting the four governmental quarters of Iceland until the 17^th^ century (^©^National and University Library of Iceland). Skriðuklaustur (blue) served the south‐eastern quadrant depicted in green. These districts were not active during the occupation of Skeljastaðir (red)

One older adult male (SKR 174) with bone changes indicative of Paget's disease (e.g., expansion of cranial diploë, endocranial “cotton wool” appearance and diffuse, irregular new bone formation throughout the cranium and all long bones) (see Ortner, 2003) had the lowest ^87^Sr/^86^Sr value, which may imply that he resided at a site further inland during childhood (see Figure 7). According to clinical research, individuals with Paget's disease involving the tibia, femur, or acetabular portion of the ilium have clinically and statistically significant functional and mobility impairments (Lyles et al., 1995). Aside from limited mobility, the condition often causes muscular diseases (e.g., atrophy) and sensory or psychological impairments (e.g., hearing loss, dementia) (Kimonis et al., 2008; Monsell, 2004) that can lead to social disability (see Roberts, 2000). This individual possibly moved to Skriðuklaustur from a site further inland for treatment or hospice care, potentially with the aid of his community. According to written documents, medieval Icelandic monasteries each served specific regions or districts of the country, meaning that people traveled to their regional monastery when in need of their services (Kristjánsdóttir, 2016; Kristjánsdóttir, 2017). In some cases, bodies were even moved from the place of death to the local monastery for proper burial and funerary services (Kristjánsdóttir, 2017, pp. 134, 248–249). While not all monasteries in Iceland served as hospitals, they were generally obligated to provide hospitality to travelers and the poor (Kristjánsdóttir, 2016; Kristjánsdóttir, 2017). As Skriðuklaustur served the south‐eastern area of the country (Kristjánsdóttir, 2016) (see Figure 7), the isotope results might suggest that some of the sampled individuals migrated there from other places within its district. On the other hand, according to the ^87^Sr/^86^Sr results of Price and Gestsdóttir (2006), only one individual (ÞSK 39) out of 33 analyzed from Skeljastaðir was of nonlocal origin. This individual also exhibits a δ^15^N value significantly higher than other individuals measured from Skeljastaðir both in this study and as reported in Sveinbjörnsdóttir et al. (2010) (see Figure 2). The difference in this individual's diet may therefore be correlated with their foreign provenance prior to their migration to Skeljastaðir.

### 5.3 Dietary reconstruction

The results of this study indicate that a primarily terrestrial protein diet was consumed at Skeljastaðir, thus confirming the results by Sveinbjörnsdóttir et al. (2010). Within a few decades of AD 1000, during the Medieval Warm Period, sea fishing in the North Atlantic increased dramatically (Barrett, Locker, & Roberts, 2004). The lack of significant marine dietary input at Skeljastaðir may possibly relate to a reduced reliance on sea fishing during the occupation of the site (1000–1104 AD). However, this may also simply relate to the long distance (~60 km) between Skeljastaðir and the coast. In parallel, using δ^13^C and δ^15^N isotope analysis on British skeletons, Müldner and Richards (2007) demonstrated that High Medieval populations consumed significantly more marine resources than populations from earlier periods and that fasting regulations imposed by the church at this time were the probable cause. Although there is no documentary evidence regarding Skeljastaðir, it is archeologically well known that up to 15 other farms successfully operated in the Þjórsárdalur valley prior to the AD 1104 eruption of Hekla. The region has ample grazing land and bodies of water filled with freshwater fish (Dugmore et al., 2007; Gestsdóttir, 2014; Steffensen, 1943; Þórðarson, 1943). When compared with the δ^13^C and δ^15^N values from bone collagen, the results of the sulfur isotope (δ^34^S) analysis suggest that some individuals at Skeljastaðir were consuming more freshwater protein than others, while those from Skriðuklaustur appear to have been consuming both saltwater and freshwater resources (see Table 7 and Figure 3b,c). At Skriðuklaustur, one individual (SKR 172) exhibits a high δ^13^C and δ^15^N with a notably lower δ^34^S value, indicating a diet with freshwater protein and possibly movement from an area further inland (see Figure 3b,c). Three individuals (SKR 14, 135, 152) show higher δ^13^C, δ^15^N, and δ^34^S values suggesting a diet high in marine protein or movement from a coastal area. Overall, the human δ^34^S values for both sites define a similar range and are consistent with ^87^Sr/^86^Sr values, indicating a population strongly impacted by marine sulfur. Furthermore, SKR 14, an adolescent, presented with a δ^13^C_carbonate_ value of −12.9‰ and δ^13^C_collagen_ value of −17.9‰ as well as the highest δ^15^N_collagen_ value (15.7‰) among the individuals sampled from Skriðuklaustur, likewise indicating a high dependency upon marine dietary resources. Similarly, one young adult male (SKR 152) appears to have maintained a marine dietary signal since childhood, exhibiting high δ^13^C_carbonate_ value, the highest δ^13^C_collagen_ value, and high δ^15^N and δ^34^S bone collagen values, implying a coastal origin. At Skeljastaðir, one male individual (ÞSK 44) showed higher δ^34^S and δ^13^C values than the others, indicating movement from a coastal area to the inland site (see Figure 3c). However, it is particularly important to consider in the context of Iceland that sulfur concentrations in flora and water sources fluctuate in response to volcanic activity (Sayle et al., 2013). One study demonstrated that due to magmatic degassing, sulfur concentrations were elevated in water sources near Skeljastaðir in the Þjórsárdalur valley even 15 years after the last eruption of Hekla (Holm et al., 2010). Although the impact of volcanic activity in the Veiðivötn–Bárðarbunga system on sulfur concentrations in flora and water near Skriðuklaustur are unknown, the severe eruption of Veiðivötn in AD 1477 caused the permanent abandonment of many of the farms in the nearby Hrafnkelsdalur valley and likely other sites in the east as well (Rafnsson, 1990, p. 93, 100). Skriðuklaustur was established just 16 years later (Kristjánsdóttir, 2016) implying that the surrounding environment may have still been considerably altered by volcanic emissions. As sulfur isotope ratios may reflect both diet and geological provenance, it is important to consider that δ^34^S values may increase in populations residing close to active, or erupting, volcanic systems.

### 5.4 Trace element analyses

The trace element analyses of dental enamel determined low Ba concentrations, which probably results from the low Ba content in seawater and Icelandic groundwater and basalt (see Naimy, 2008) (see Supplementary Figure S1). The low values, small range, and little variation in Ba concentrations also corroborate the interpretation that the people residing at Skriðuklaustur represented a local population of individuals that grew up in Iceland. The means for Pb indicate that at least some anthropogenic exposure to lead occurred at Skriðuklaustur (Supplementary Figures S1 and S2). Only 12 individuals exhibited Pb concentrations greater than ~0.7 ppm (see Table 7), which is normally considered the threshold in archaeological human dental enamel between natural and anthropogenic Pb exposure (Millard et al., 2014; Montgomery et al., 2010). All the individuals with anthropogenically elevated lead levels are nonadults or are biologically female except for the older adult male with Paget's disease (SKR 174), potentially indicating differential exposure related to gendered social roles or the life course. Lead exposure may occur from dermatological contact with lead objects or structures, inhalation of lead dust or paint chips or the ingestion of food, soil, or other substances contaminated with lead dust. Women generally spent more time indoors, tasked with maintaining the household, food preparation and weaving textiles that were used both as clothing and currency (*vaðmál*). Due to the importance of cloth currency for its use to pay tithes, taxes and other legal or economic transactions, cloth production was intensely standardized. Some evidence even suggests that legal controls may have regulated occupational roles and simultaneously that cloth currency production was also an important form of female agency to Icelandic society (Norrman, 2008; Smith, 2014). Additionally, the accumulation, retention, and susceptibility to the health effects of exposure to toxic metals have been demonstrated to differ between men and women, particularly during pregnancy or menopause (Vahter, Åkesson, Lidén, Ceccatelli, & Berglund, 2007). Regarding children, it is well established that they not only absorb a far greater amount of ingested lead than adults but are also more frequently exposed to it due to hand‐to‐mouth activities and other behavioral tendencies such as outdoor play (see Jacobs & Nevin, 2006; Wittmers, Aufderheide, Rapp, & Alich, 2002). Women and children may have been more regularly exposed to lead at home due to more frequent contact with lead objects and structures within the household. A young adult female (SKR 189) with cystic echinococcosis (hydatid disease) had a Pb concentration of 9.40 ppm, significantly higher than all others in the sample set, indicating substantial childhood anthropogenic exposure to lead (see Montgomery et al., 2010). The individual (SKR 23) with the second highest Pb concentration (4.1 ppm) was a young adult (c. 17–25) female with treponemal disease, exhibiting skeletal changes consistent with venereal syphilis, according to the criteria described by Hackett (1976) and Ortner (2003). Clinical research has also demonstrated that deficiencies in essential minerals, such as calcium, can result in the abnormal and rapid absorption of toxic heavy metals or trace elements, such as Pb, particularly in malnourished children (Talpur, Afridi, Kazi, & Talpur, 2018). It is thereby possible that some individuals with elevated lead concentrations may have had low calcium intake during childhood. It is evident that parts of the cemetery were contaminated with anthropogenic lead, which is likely to be associated with the infrastructure of the monastery (e.g., lead window frames; door hinges) and objects found on site (Kristjánsdóttir, 2012). By the 17th century, lead‐glazed kitchenware significantly increased in availability in Iceland, particularly among high status individuals, but it has also been found at sites dating to the early medieval period (Þorgeirsdóttir, 2010). For context, Rasmussen, Skytte, Jensen, and Boldsen (2015) measured Pb concentrations in individuals that resided in rural, monastic, and urban sites around Denmark and northern Germany. Their results indicate that higher status, urban dwellers were more likely to live among lead structures (e.g., window frames, roof tiles possibly in contact with drinking water) and be able to afford lead or lead‐glazed kitchenware. Another female (SKR 65) exhibited a Pb concentration of 3.51 ppm. She was one of a few individuals found buried within the church itself, potentially indicating that she was a benefactor or had a special status at the monastery (Kristjánsdóttir, 2010; Walser et al., 2018). It is therefore possible that the individuals with elevated anthropogenic Pb concentrations were exposed to lead within their households or the monastic grounds, if they resided there during childhood. Nonetheless, with a small number of exceptions the range of Pb concentrations determined in dental enamel is largely below ~0.7 ppm, suggesting that those analyzed from Skriðuklaustur represent individuals that grew up in an unpolluted environment, such as Iceland (see Montgomery et al., 2014).

The highest Zn concentration was 145.8 ppm (SKR 10), which is still well within the lower spectrum of expected Zn concentrations in dental enamel (9.9–1,550 ppm) (see Jaouen, Herrsher, & Balter, 2017). Clinical studies have noted a relationship between malnutrition and lower enamel zinc concentrations (Brown et al., 2004) and that enamel Zn concentrations of <90 ppm may reflect marginal zinc supply during childhood (e.g., Tvinnereim et al., 1999). The individuals with the lowest zinc concentrations include an adult female (SKR 195) (43.8 ppm) and an adult male (SKR 150) (47.3 ppm), possibly implying limited zinc supply during childhood (see Supplementary Figure S2). These two individuals also exhibit dental enamel hypoplasia, a pathological indicator of metabolic or health stress during childhood (see Ortner, 2003). However, zinc is an essential trace element under homeostatic control and its concentrations are altered by numerous and complex interactions including diet, disease, individual variation, digestion, and absorption. As a result, zinc concentrations determined in dental enamel may not accurately reflect palaeodiet (Dolphin & Goodman, 2009; Ezzo, 1994).

## 6 CONCLUSIONS

The skeletons sampled from Skriðuklaustur appear to represent individuals from the geographical region (south‐east quarter of Iceland) served by the monastery. The ^87^Sr/^86^Sr, δ^13^C_carbonate_, and δ^18^O_phosphate_ and trace element concentrations (Pb, Ba, Zn, Sr) results indicate that the individuals analyzed in this study were likely to have been born in Iceland but the clear positive correlation between ^87^Sr/^86^Sr, δ^13^C_carbonate_, and Sr concentrations indicates that some had lived inland during childhood and some closer to or at the coast. Additionally, no significant differences between males and females were observed. There is thus no evidence that imported foodstuffs were shifting the human ^87^Sr/^86^Sr outside the Icelandic range. This research also provides the first dataset for inferred drinking water (δ^18^O_dw_) values determined from human dental enamel from Iceland. The δ^18^O_dw_ values range from −12.3 to −8.9‰, with a mean of −10.2 ± 1.0‰, which fit within the annual δ^18^O for precipitation in Iceland (−13 to −8‰) (see Price et al., 2015, figure 20; Bowen, 2018). The enamel Ba and Pb concentrations (ppm) were low, probably due to the low concentrations of these elements in the Icelandic environment and saltwater, which may corroborate the conclusion of Icelandic origins of those buried at Skriðuklaustur. These individuals may have sought medical treatment or hospice at the monastery, as was common in Iceland at the time (see Kristjánsdóttir, 2017). Despite the over 350 year time difference, the δ^13^C and δ^15^N values determined in bone collagen indicate that the individuals residing at Skriðuklaustur consumed a diet high in marine protein, while those residing at Skeljastaðir exhibit values more consistent with reliance on terrestrial resources. No significant differences between men and women were observed at either site. When δ^13^C and δ^15^N values are compared with the δ^34^S values, the results indicate that three individuals from Skriðuklaustur and one individual from Skeljastaðir may have migrated from coastal sites or areas heavily affected by sea spray. The δ^34^S values also imply freshwater fish consumption at both sites. At least one individual from the Skriðuklaustur assemblage consumed a different diet than others residing at the monastery, potentially due to the pathological conditions they were suffering from. Three individuals from Skriðuklaustur may have experienced a period of illness or metabolic stress during childhood according to their low Zn concentrations and the presence of linear enamel hypoplasia. Finally, approximately 12 individuals from Skriðuklaustur may have been exposed to anthropogenic sources of lead during childhood (Pb concentrations >0.7 ppm), possibly from the lead objects and window frames that were found during the excavation of the monastery ruins.

Overall, the dietary differences noted between the two noncontemporary inland sites may reflect cultural changes in trade, subsistence strategies, and environment (e.g., the Little Ice Age, volcanic eruptions) in medieval Iceland. Furthermore, the results of isotope analyses conducted on individuals excavated from Skriðuklaustur indicates that the monastery was operated, visited and inhabited by the local population of brethren, pilgrims, patients, and other local individuals. Considering the functions of the monastery, these findings also provide further evidence for the movement of disease (e.g., syphilis), goods (e.g., lead wares), food (e.g., fish, fruit), and information (e.g., medicine) from other parts of Iceland and abroad, implying that these past people were never found in isolation, even at the edge of the world.

## CONFLICT OF INTEREST

The authors declare no potential conflict of interest.

## Supporting information


**Appendix S1**: Supporting Information

**Supplementary Figure S1** Chart demonstrating the Pb and Ba concentrations (ppm) among individuals sampled from Skriðuklaustur.

**Supplementary Figure S2** Chart demonstrating the Pb and Zn concentrations (ppm) among individuals sampled from Skriðuklaustur.

## Data Availability

The authors confirm that the data supporting the findings of this study are available within the article [and/or] its supplementary materials.
