# Supplementary material for: At the world's edge: Reconstructing diet and geographic origins in medieval Iceland using isotope and trace element analyses
Source: Am J Phys Anthropol. 2019 Dec 13;171(1):142–63. doi: 10.1002/ajpa.23973 (PMC6973133; doi:10.1002/ajpa.23973)
Supplement: Supplementary file 1 — Appendix S1: Supporting Information [file AJPA-171-142-s001.docx]

**Supplementary Materials**

*Carbon and nitrogen isotope analysis*

Bone collagen was extracted using the modified Longin method (see Brown, Nelson, Vogel, & Southon, 1988). Total organic carbon, total nitrogen content and stable isotope analysis of the samples were performed using a Costech Elemental Analyser (ECS 4010) connected to a ThermoFinnigan Delta V Advantage isotope ratio mass spectrometer. Carbon isotope ratios were corrected for ^17^O contribution and reported in standard delta (δ) notation in per mil (‰) relative to Vienna Pee Dee Belemnite (VPDB). Isotopic accuracy was monitored through routine analyses of in-house standards, which were stringently calibrated against international standards (e.g., USGS 40, USGS 24, IAEA 600, IAEA N1, IAEA N2): this provided a linear range in δ^13^C between –46 ‰ and +3 ‰ and in δ^15^N between –4.5 ‰ and +20.4 ‰. Analytical uncertainty in carbon and nitrogen isotope analysis was typically ±0.1 ‰ for replicate analyses of the international standards and typically <0.2 ‰ on replicate sample analysis. Total organic carbon and nitrogen data was obtained as part of the isotopic analysis using an internal standard (Glutamic Acid, 40.82 % C, 9.52 % N).

*Sulphur isotope analysis*

Sulphur isotopic analysis of collagen samples were performed using a Costech Elemental Analyser (ECS 4010) connected to a Thermo Scientific Delta V Plus isotope ratio mass spectrometer. Collagen was weighed out into 10x10mm tin capsules (between 4–6mg) and approximately the same weight of vanadium pentoxide (V_2_O_5_) was added to aid in the combustion process to release sulphur. Isotopic accuracy was monitored using the following international sulphur standards: IAEA-S-2, IAEA-S-3, IAEA-S-4, IAEA-SO-5, and NBS 127. Analytical uncertainty in sulphur isotope analysis was typically <0.2 ‰ for replicate analyses of the international standards. Total sulphur was obtained as part of the isotopic analysis using the international standards listed above.

*Enamel carbon and oxygen isotope analysis*

Carbon(δ^13^C) and oxygen (δ^18^O) isotope ratios were measured in the carbonate (CO_3_) component of tooth enamel. For each tooth, approximately 2mg of powdered sample was weighed and transferred into an individual exetainer vial. Vials were flushed with helium (grade 4.5) then CO2 was liberated by reaction with 99% ortho-phosphoric acid for 2 hours at 70^o^C. The resultant gas mix of helium and CO_2_ was transferred through a Thermo Fisher Scientific Gasbench II in which a gas chromatographic column separated the CO_2_ from the gas mixture then passed into a Thermo Fisher Scientific MAT 253 gas source mass spectrometer for isotopic analysis.

The following international reference materials were analysed with each batch of samples: NBS 18 (calcite, *n*=3), IAEA-CO-1 (marble, *n*=3) and LSVEC (Lithium Carbonate, *n*=3). In addition, two internal standards: DCS01 (calcium carbonate, *n*=6) and Dobbins (horse tooth, *n*=2) were also analysed. Repeated analysis of both international and internal standards yielded an analytical precision of 0.20‰ (2 s.d.) for δ^13^C and 0.24‰ (2 s.d.) for δ^18^O. Duplicate analyses of the same sample reproduced within or better than 0.06‰ and corrections were made using IAEA-CO-1 and LSVEC, with all δ^13^C and δ^18^O values reported relative to the Vienna PeeDee Belemnite (VPDB) standard. δ^18^O was additionally reported relative to the Vienna Standard Mean Ocean Water (vsmow) standard for comparison purposes.

*Enamel strontium isotope analysis*

The enamel samples were collected following the procedure given in Montgomery (2002). For each molar, a single chip of enamel weighing approximately 20mg was collected using a diamond-tipped rotary dental saw. All surfaces of the enamel samples were cleaned and polished with a diamond-tipped dental burr to a depth of >100 μm to remove traces of contaminants such as soil and dentine. Cleaned enamel samples were analysed in the Arthur Holmes Isotope Geology laboratory, Department of Earth Sciences, Durham University using column chemistry methods outlined in Font *et al*. (2008). Samples were first dissolved in 3M HNO_3_ and heated overnight on a hot plate. The samples were loaded onto cleansed and preconditioned columns containing 60μl of strontium-specific resin. 2x250 μl 3M HNO_3_ was passed through to elute the waste, then 2x200 μl MQ H_2_O was passed through to elute the strontium, which was collected. 17μl of ~15.5M HNO3 was added to the Sr fraction to make the solution 3% HNO_3_. Following preparation, the size of the ^86^Sr beam was tested for each sample to assess the strontium concentrations. From this analysis, a dilution factor could be calculated for each sample and each was diluted to yield a beam size of approximately 20V ^88^Sr, where possible, to match the beam size of the isotope reference material, NBS987. The strontium samples were analysed by Multi-Collector Inductively Coupled Plasma Mass Spectrometry (MC-ICP-MS) using a Neptune MC-ICP-MS. Samples were introduced into this using an ESI PFA50 nebuliser and a glass expansion cinnabar micro-cyclonic spray chamber. Instrumental mass bias was corrected for using an ^88^Sr/^86^Sr ratio of 8.375209 (the reciprocal of the accepted ^86^Sr/^88^Sr ratio of 0.1194) and an exponential mass fractionation law. Corrections were also applied for Kr interferences on ^84^Sr and ^86^Sr, derived from Ar gas supply, and the Rb interference on ^87^Sr, derived from the sample, by monitoring masses ^82^Kr, ^83^Kr and ^85^Rb respectively. The average ^83^Kr intensity throughout the analytical session was ~0.22mV, which is insignificant considering the Sr beam size (^88^Sr between 4.6 and 27V, average of 14.8V). The average ^85^Rb intensity was slightly greater at ~0.61mV (range: 0.20-1.1 mV) but again, given the range in Sr beam size, the Rb correction on the ^87^Sr/^86^Sr, was very small (<0.00001) and is accurate at that magnitude. The samples were analysed in two analytical sessions (see Table 9 for details) during which the average ^87^Sr/^86^Sr value and reproducibility for the international isotope reference material NBS987 was 0.710258±0.000013 (2SD; n=12) and 0.710266±0.000009 (2SD; n=6). Data in Table 9 are renormalized to an accepted value for NBS 987 of 0.71024. Total procedural Sr blanks run during the period of study were <100pg, which is insignificant relative to the average sample size of 143ng (0.07% blank) and even the minimum sample size of 26ng (0.38% blank) and do not require application of a blank correction.

*Trace element analysis*

In a 1.5ml plastic vial the pre-weighed sample (5-10mg) had 1ml of 3N HNO3 added and was left overnight to dissolve. 0.5ml was then transferred to a 15ml autosampler vial and diluted to 10ml. Samples were analysed by ICP-MS (Thermo Scientific XSeries2) previously optimised for low oxide and double charge interferences and calibrated for Sr, Ba, Zn and Pb. Calibration standards and blank were analysed throughout the sample sequence to monitor and correct for any instrumental drift. Final enamel concentration was then determined based on sample weight and total dilution volume. Reanalysing the same sample from the 15ml vial is reproducible ±2%.

*Supplementary Figure Captions*

Supplementary Figure 1. Chart demonstrating the Pb and Ba concentrations (ppm) amongst individuals sampled from Skriðuklaustur.

Supplementary Figure 2. Chart demonstrating the Pb and Zn concentrations (ppm) amongst individuals sampled from Skriðuklaustur.
