# Supplementary figures and images for: At the world's edge: Reconstructing diet and geographic origins in medieval Iceland using isotope and trace element analyses
Source: Am J Phys Anthropol. 2019 Dec 13;171(1):142–63. doi: 10.1002/ajpa.23973 (PMC6973133; doi:10.1002/ajpa.23973)

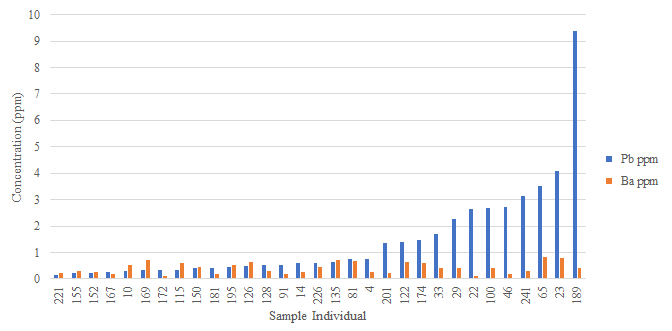

Supplement: Supplementary file 2 — Supplementary Figure S1 Chart demonstrating the Pb and Ba concentrations (ppm) among individuals sampled from Skriðuklaustur. [file AJPA-171-142-s002.tif]

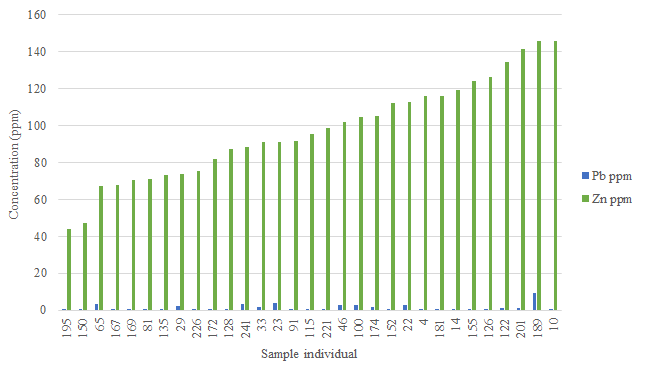

Supplement: Supplementary file 3 — Supplementary Figure S2 Chart demonstrating the Pb and Zn concentrations (ppm) among individuals sampled from Skriðuklaustur. [file AJPA-171-142-s003.tif]
